# The histone demethylase dKDM5/LID interacts with the SIN3 histone deacetylase complex and shares functional similarities with SIN3

**DOI:** 10.1186/s13072-016-0053-9

**Published:** 2016-02-03

**Authors:** Ambikai Gajan, Valerie L. Barnes, Mengying Liu, Nirmalya Saha, Lori A. Pile

**Affiliations:** Department of Biological Sciences, Wayne State University, Detroit, MI USA

**Keywords:** Histone demethylase, SIN3 complex, Cell proliferation, Wing development, Gene expression, RNAseq, Oxidative stress, *Drosophila*

## Abstract

**Background:**

Regulation of gene expression by histone-modifying enzymes is essential to control cell fate decisions and developmental processes. Two histone-modifying enzymes, RPD3, a deacetylase, and dKDM5/LID, a demethylase, are present in a single complex, coordinated through the SIN3 scaffold protein. While the SIN3 complex has been demonstrated to have functional histone deacetylase activity, the role of the demethylase dKDM5/LID as part of the complex has not been investigated.

**Results:**

Here, we analyzed the developmental and transcriptional activities of dKDM5/LID in relation to SIN3. Knockdown of either *Sin3A* or *lid* resulted in decreased cell proliferation in S2 cells and wing imaginal discs. Conditional knockdown of either *Sin3A* or *lid* resulted in flies that displayed wing developmental defects. Interestingly, overexpression of dKDM5/LID rescued the wing developmental defect due to reduced levels of SIN3 in female flies, indicating a major role for dKDM5/LID in cooperation with SIN3 during development. Together, these observed phenotypes strongly suggest that dKDM5/LID as part of the SIN3 complex can impact previously uncharacterized transcriptional networks. Transcriptome analysis revealed that SIN3 and dKDM5/LID regulate many common genes. While several genes implicated in cell cycle and wing developmental pathways were affected upon altering the level of these chromatin factors, a significant affect was also observed on genes required to mount an effective stress response. Further, under conditions of induced oxidative stress, reduction of SIN3 and/or dKDM5/LID altered the expression of a greater number of genes involved in cell cycle-related processes relative to normal conditions. This highlights an important role for SIN3 and dKDM5/LID proteins to maintain proper progression through the cell cycle in environments of cellular stress. Further, we find that target genes are bound by both SIN3 and dKDM5/LID, however, histone acetylation, not methylation, plays a predominant role in gene regulation by the SIN3 complex.

**Conclusions:**

We have provided genetic evidence to demonstrate functional cooperation between the histone demethylase dKDM5/LID and SIN3. Biochemical and transcriptome data further support functional links between these proteins. Together, the data provide a solid framework for analyzing the gene regulatory pathways through which SIN3 and dKDM5/LID control diverse biological processes in the organism.

**Electronic supplementary material:**

The online version of this article (doi:10.1186/s13072-016-0053-9) contains supplementary material, which is available to authorized users.

## Background

Chromatin, composed of DNA wrapped around histone proteins, acts as the template for gene transcription in eukaryotes. The activity of nucleosome remodeling factors and histone-modifying enzymes, as well as the incorporation of histone variants, regulates dynamics of chromatin packaging [1]. Dense packaging of chromatin is associated with transcription repression, while a more loose conformation is associated with activation. Histone-modifying enzymes regulate transcription by modifying the N-terminal tails of histones, enabling or preventing the association of several distinct transcriptional activators and repressors [2]. Analysis of many immunopurified chromatin regulatory complexes indicates the presence of multiple histone-modifying enzymes within the same complex. Two such enzymes found to occur together in chromatin regulatory complexes are histone deacetylases (HDACs) and lysine demethylases (KDMs), reviewed by Hayakawa and Nakayama [3]. The SIN3, NuRD, CoREST, and NCoR/SMRT complexes have all been shown to include both class I HDACs and a KDM.

The activity of HDACs typically leads to a transcriptionally repressive chromatin environment while the opposing activity of lysine acetyltransferases (KATs) results in an environment favorable for transcription [2]. SIN3 acts as a scaffold protein for multiple HDAC complexes present in organisms from yeast to mammals and is thus generally associated with transcription repression [4]. Across species, the distinct SIN3 complexes share much similarity in composition of proteins and biological functions. The HDAC RPD3, in yeast and *Drosophila*, and HDAC1 and 2, in mammals, render catalytic activity to the complex. Investigations using different model organisms have identified the interaction of SIN3 with a KDM, dKDM5/LID, in *Drosophila,* and the homolog KDM5A in mammals [5–8]. This finding adds a second catalytic component to the SIN3 complex, which to date had been regarded as an HDAC complex. In *Drosophila*, a single gene, *Sin3A*, encodes multiple isoforms of SIN3. Work in our laboratory has shown that dKDM5/LID predominantly associates with the largest SIN3 isoform, SIN3 220 [7].

*Sin3A* is an essential gene in both *Drosophila* and mammals [9–13]. SIN3 was initially identified in yeast as a global regulator of transcription [14, 15]. In *Drosophila*, microarray expression analysis of S2 and Kc cultured cells upon *Sin3A* RNA interference (RNAi), determined that ~3 % of the genome is regulated by SIN3, where a vast majority of genes were repressed by SIN3 [16]. Further, SIN3 plays an important role in cell cycle progression. In *Drosophila*, knockdown of *Sin3A* by RNAi in cultured cells leads to a G2/M phase cell cycle arrest [17]. In mammals, two distinct genes *mSin3a* and *mSin3b,* encode SIN3 proteins. In mouse embryonic fibroblasts (MEFs), deficiency of mSIN3A leads to a reduction in proliferative capacity and an increase of cells in the G2/M phase of the cell cycle [9, 10]. mSIN3B-deficient MEFs, however, continue to proliferate, but fail to exit the cell cycle [11]. Furthermore, SIN3 is known to be important for developmental processes. In *Drosophila*, SIN3 isoforms show differential expression in multiple tissues and life stages [18]. Knockdown of *Sin3A* at different developmental time points indicates a requirement for SIN3 during multiple stages of development [18, 19]. SIN3 is also linked to key developmental and signaling pathways. SIN3 is associated with steroid hormone, Notch, ERK and JNK signaling pathways [20–24]. SIN3 is further implicated in eye, wing, neural and cardiac development [12, 25–28].

Similar to *Sin3A*, *lid* is an essential gene in *Drosophila,* first identified in a screen for *trithorax* group genes [29]. dKDM5/LID is a JmjC domain containing KDM, which specifically removes H3K4me3, a mark associated with active transcription [30–33]. In mammals, four paralogous genes encode *lid* homologs, KDM5A through KDM5D. KDM5A, KDM5B, and KDM5C interact with SIN3 or HDAC complexes [5, 8, 34–36]. Until recently, targeted gene expression analysis had been performed for only a few genes to understand the role of dKDM5/LID in transcription. These studies revealed that, consistent with its demethylase activity, Notch target genes are repressed by dKDM5/LID, while other genes are positively regulated [6, 31–33, 37]. Recently, two groups published findings for genome-wide changes in gene expression upon loss or reduction of dKDM5/LID [38, 39]. These groups utilized expression arrays to identify dKDM5/LID-regulated genes in *Drosophila* wing imaginal disc tissues. Work by Lloret-Llinares et al., while demonstrating that a large number of genes are bound by dKDM5/LID, identified very few genes that showed statistically significant changes in expression [39]. In contrast, Liu et al. reported a large number of genes (901) to be regulated by dKDM5/LID, of which 367 were upregulated and 534 were downregulated, suggesting a role in both gene activation and repression [38]. Additionally, dKDM5/LID is necessary for fly development [29, 40]. In mammals, similar to SIN3, KDM5A and KDM5B are known to regulate cell cycle progression [34, 41–45].

Taken together, research in *Drosophila* and mouse suggests that SIN3 and KDM5 have overlapping as well as distinct biological functions. In this work, we wished to further explore the potential intersection of functional activities of these two transcriptional regulators. Here, we have focused on the role of dKDM5/LID in the context of the *Drosophila* SIN3 complex. We demonstrated that dKDM5/LID acts similarly to SIN3 in affecting cell cycle progression and wing development, strongly supportive of a functional interaction of these proteins. Through genome-wide expression analysis we determined that SIN3 and dKDM5/LID regulate transcription of a number of common gene targets with specific effect on stress tolerance processes. Further, we found that under conditions of oxidative stress, SIN3 and dKDM5/LID proteins affect a large number of genes implicated in cell cycle control.

## Results and discussion

### SIN3 and RPD3 co-purify with dKDM5/LID

**Fig. 1:**
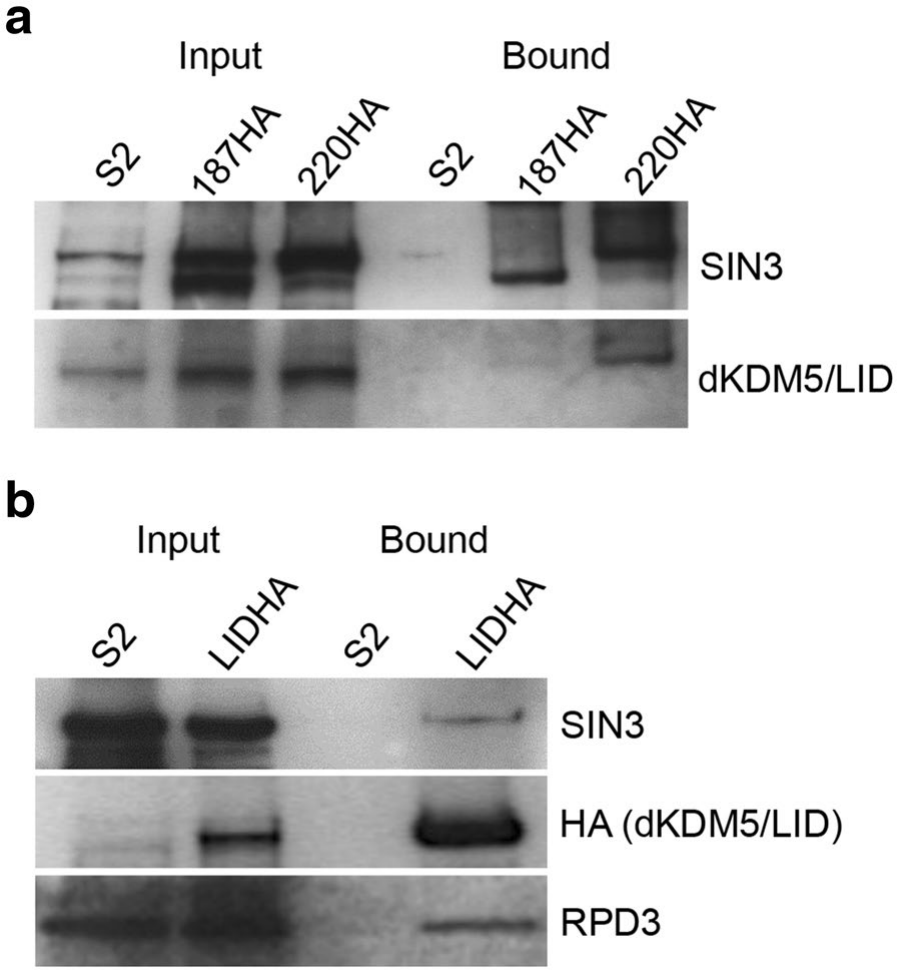
dKDM5/LID interacts with SIN3 complex components. Western blot analysis of input and bound fractions of nuclear extracts from S2, SIN3 187HA, and SIN3 220HA (**a**) or S2 and dKDM5/LID FLAG-HA (**b**) cells. Antibody to the HA tag was used for immunoprecipitation. Blots were probed with the antibody listed to the *right*

We previously identified components of *Drosophila* SIN3 187 or 220 isoform specific complexes by LC/MS/MS analysis and determined that dKDM5/LID co-purifies predominantly with the SIN3 220 complex [7]. To build on that study, we sought to analyze the interaction of dKDM5/LID with SIN3 220 by western blot assay using dKDM5/LID-specific antibody. Nuclear extracts were prepared from S2 cells and cells expressing HA-tagged SIN3 187 or 220. Nuclear extracts were subjected to immunopurification of SIN3 using anti-HA beads. Western blot with antibody to dKDM5/LID or antibody to SIN3 showed the association of dKDM5/LID predominantly with SIN3 220 (Fig. 1a). To validate the interaction of dKDM5/LID with SIN3, we performed the reciprocal experiment, where we immunopurified dKDM5/LID. We generated a *Drosophila* S2 cell line carrying a transgene for inducible expression of FLAG-HA-tagged dKDM5/LID. We immunopurified nuclear extracts prepared from control S2 cells and dKDM5/LID FLAG-HA cells using anti-HA beads. A western blot of immunoprecipitated dKDM5/LID showed interaction of dKDM5/LID FLAG-HA with SIN3 and RPD3, two components of the SIN3 complex (Fig. 1b). As an additional control, we immunopurified nuclear extracts prepared from S2 cells, cells expressing HA-tagged SIN3 187 or 220, and cells expressing FLAG-HA-tagged dKDM5/LID with IgG and probed with antibody to HA. No detectable levels of HA-tagged proteins were present in the bound fraction (data not shown). Previously two other groups purified dKDM5/LID and demonstrated its interaction with components of the SIN3 complex [6, 31]. Work by Lee et al., demonstrated an association of dKDM5/LID with RPD3 and Pf1, another component of the SIN3 complex, but did not find an interaction with SIN3 itself [31]. However, Moshkin et al., isolated a dKDM5/LID complex that includes SIN3 and RPD3 proteins as well as Pf1 and EMSY, components of the SIN3 complex [6]. Our work along with published data establishes the interaction of dKDM5/LID with a SIN3 and RPD3 containing complex.

### dKDM5/LID affects cell cycle progression

Having verified the interaction of dKDM5/LID with SIN3 and the catalytic component of the complex, the HDAC RPD3, we sought to determine the contribution of dKDM5/LID to SIN3 complex functions. Previous work in yeast, flies, and mammals has shown SIN3 to play an important role in regulating progression through the cell cycle [9, 11, 17, 46]. In *Drosophila,* in addition to SIN3, other components of the SIN3 complex such as RPD3 and p55 have also been shown to affect cell growth rates of cultured cells [17]. We next investigated if dKDM5/LID also contributed to progression through the cell cycle.

**Fig. 2:**
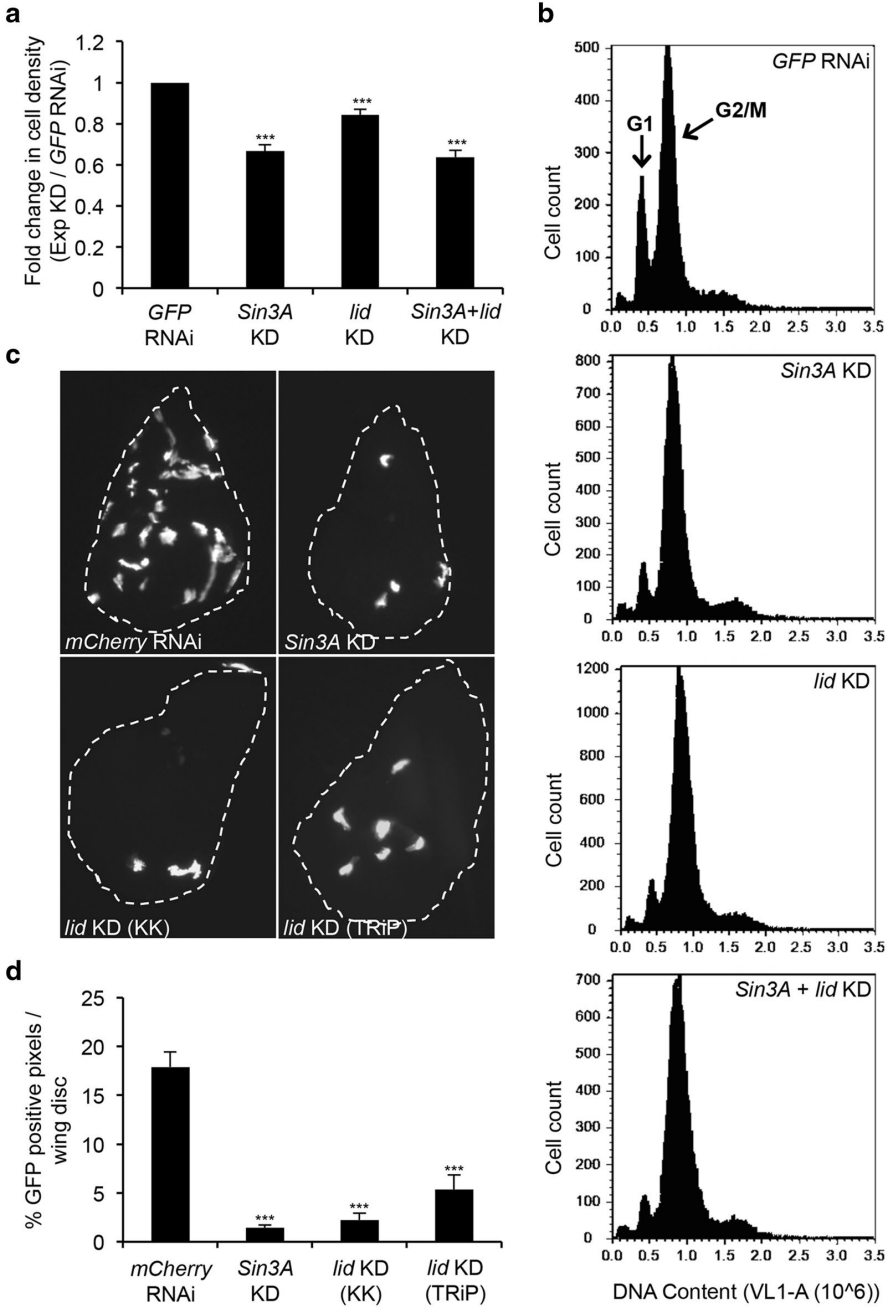
Knock down of *lid* leads to decreased cell proliferation in S2 cells and wing imaginal discs, similar to a reduction of *Sin3A.* **a** Quantification of cell density by cell counts of S2 cells treated with dsRNA targeting indicated proteins. Results are the average of five biological replicates. **b** Histogram of DNA content vs. cell counts for indicated cell lines by flow cytometry. Peaks for G1 and G2/M cell cycle phases are indicated in the *top* *panel*. **c** Control and *Sin3A* or *lid* knockdown wing disc clones generated using the Flip-out GAL4 system and immunostained with antibody to GFP. **d** Quantification of GFP signal in wing imaginal discs. Results are the average pixel counts from 20 wing imaginal discs. *KD* knockdown. ***, *P* < 0.001

First, we checked for defects in cell proliferation by measuring cell density of *Drosophila* S2 cells upon induction of RNAi of *Sin3A*, *lid*, or both. S2 cells treated with dsRNA targeting *GFP* was used as a control. Verification of efficient reduction of SIN3 protein by western blot analysis and *lid* transcript by real-time quantitative RT-PCR (qRT-PCR) is routinely performed in the lab (Additional file 1: Figure S1). Determination of cell density revealed that *lid* knockdown cells had decreased cell density, about 15 % lower, compared to control cells treated with *GFP* dsRNA (Fig. 2a). *lid* knockdown, however, resulted in a less severe cell proliferation defect compared to *Sin3A* knockdown cells. Further, double knockdown of *lid* and *Sin3A* did not result in an additive effect on cell proliferation. The double knockdown cells showed densities comparable with single knockdown of *Sin3A*. These results suggest that multiple components of the SIN3 complex could contribute to the cell proliferation defect seen upon loss of SIN3. Loss of the scaffold protein, SIN3, may result in the disruption of function of these additional complex components, including dKDM5/LID, thereby resulting in a more pronounced proliferation defect relative to the other components.

Next, to look at the cell cycle distribution of S2 cells upon knockdown of *Sin3A*, *lid*, or both, we performed flow cytometry analysis. Consistent with previous reports [17], *Sin3A* knockdown resulted in a G2/M phase arrest or delay of the cell cycle (Fig. 2b). Similar to the effect of *Sin3A* knockdown, *lid* knockdown and double knockdown of *Sin3A* and *lid* resulted in an accumulation of cells in the G2/M phase of the cell cycle while the number of cells in G1 was reduced compared to GFP RNAi control cells. These results further validate a role for both SIN3 and dKDM5/LID in regulating cell cycle progression.

To analyze the role of dKDM5/LID in cell proliferation in the context of fly development, we looked at clonal cell growth in *Drosophila* wing imaginal discs. For this purpose, we utilized the heat shock flip-out system to randomly generate EGFP-marked clones with or without *lid* knockdown. We previously showed that reduction of SIN3 results in clones that are few in number [28]. Similar to our previous observation with *Sin3A* knockdown, we found that reduction of dKDM5/LID also resulted in EGFP-positive clones that were fewer in number relative to the control (*mCherry* RNAi) (Fig. 2c). Quantification of the GFP-positive pixels per disc shows a three to sevenfold reduction in the number of pixels compared to the control upon *lid* knockdown (Fig. 2d). We utilized two separate RNAi lines to drive *lid* knockdown and observed similar results, which strongly suggests that the reduction in clonal cell growth is due to *lid* knockdown and not an off-target effect of RNAi. Data from both cell culture and developing flies demonstrate that dKDM5/LID plays an important role in regulating cell proliferation. The observed defects in cell proliferation in the developing wing imaginal disc cells are much more pronounced compared to cultured cells. This is possibly due to the more significant roles of SIN3 and dKDM5/LID proteins during development.

### dKDM5/LID functions in wing development

Next, we tested the role of dKDM5/LID in the regulation of developmental processes. *Sin3A* and *lid* are both essential genes in *Drosophila* and are implicated in the regulation of developmental processes [12, 13, 18, 25–29, 40]. In *Drosophila,* the GAL4-UAS system can be used to induce RNAi of target genes. Crossing an *Act*-*GAL4* driver line to a UAS-RNAi line results in progeny with ubiquitous knockdown of the gene of interest. Ubiquitous knockdown of *Sin3A* by RNAi results in lethality [28]. *lid* knockdown using a ubiquitous *Act*-*GAL4* driver was shown to result in semi lethality [30]. In our hands, upon ubiquitous knockdown of *lid* using an *Act*-*GAL4* driver, we observed varying degrees of lethality depending on the UAS-RNAi line and the temperature at which the flies were reared (Table 1). When reared at 25 °C, crosses using the *UAS*-*LID*^*RNAi*-*KK*^ line resulted in 50–70 % lethality of the progeny. At 27 °C, however, very few flies survived compared to those reared at 25 °C. Use of the *UAS*-*LID*^*RNAi*-*TRiP*^ line resulted in very few survivors, even at 25 °C and complete lethality at 27 °C. The varying degrees of lethality is possibly due to the differences in RNAi efficiency of the dsRNA constructs utilized and the temperature dependence of GAL4 activity [47]. Additionally, we note that the different LID RNAi lines produced varying degrees of the observed phenotypes. For lethality, the TRiP line gave the stronger result compared to the KK line (Table 1), while for clonal cell growth, the KK line yielded a more severe phenotype relative to the TRiP line (Fig. 2c, d). These differences are possibly due to distinct efficiency of knockdown generated using the two separate GAL4 driver lines.

**Table 1:** Ubiquitous reduction or overexpression of dKDM5/LID results in a loss of viability

		♂			♀		
		Adult progeny observed		% Viable (*UAS*-*X/* *Act*-*GAL4*)	Adult progeny observed		% Viable (*UAS*-*X/* *Act*-*GAL4*)
Temp (°C)	UAS line	(*UAS*-*X/Act*-*GAL4*)	(*UAS*-*X/CyO*)		(*UAS*-*X/Act*-*GAL4*)	(*UAS*-*X/CyO*)	
25	*UAS*-*LID* ^*RNAi*-*KK*(2)^	41	180	17.3 ± 13.3	90	165	49.6 ± 9.5
	*UAS*-*LID* ^*RNAi*-*TRiP*(1)^	0	48	0	1	53	1.9
27	*UAS*-*LID* ^*RNAi*-*KK*(2)^	0	54	0	5	63	13.5 ± 9.6
	*UAS*-*LID* ^*RNAi*-*TRiP*(3)^	0	168	0	0	237	0
	*UAS*-*LID* ^(4)^	0	228	0	0	194	0
	*UAS*-*LID*-JmjC* ^(1)^	0	63	0	0	52	0

UAS-RNAi or UAS overexpression fly lines for *lid* were crossed with the *Act*-*GAL4/CyO* driver line and the progeny were analyzed and counted

^(n)^Number of trials

*X* genotype, *KD* knockdown, *KK* RNAi line from Vienna *Drosophila* RNAi Center, *TRiP* RNAi line from Bloomington Stock Center

**Fig. 3:**
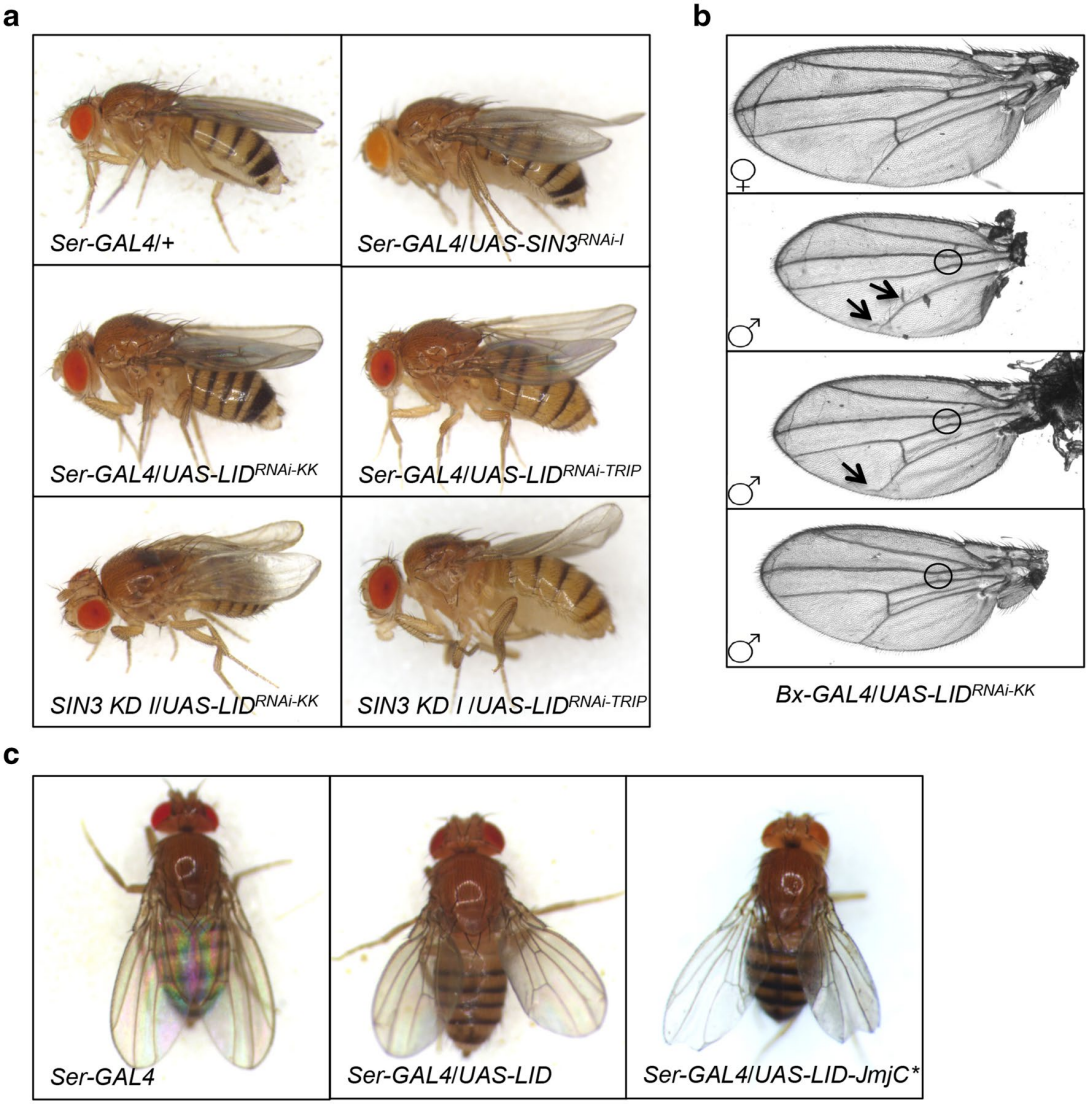
Reduction or overexpression of dKDM5/LID results in defects in wing development. **a** Micrographs of female flies carrying the *Ser*-*GAL4* driver and/or the indicated UAS-RNAi constructs. *SIN3 KD I* flies carry both the *Ser*-*GAL4* driver and *UAS*-*SIN3* ^*RNAi*^ construct. **b** Micrographs of wings from flies carrying the *Bx*-*GAL4* driver and the *UAS*-*LID* ^*RNAi*-*KK*^ construct. *Top* *panel* represents female wings showing wild-type wing venation. Male wings show varying degrees of vein disruptions, indicated by *circles* and *arrows*. **c** Micrographs of female flies carrying the *Ser*-*GAL4* driver and the indicated UAS overexpression constructs

Such lethality observed upon knockdown of either SIN3 or dKDM5/LID proteins render it necessary to utilize conditional knockdown systems to study the functional roles of these proteins during development. We previously demonstrated that knockdown of *Sin3A* in wing precursor cells results in a curved rather than straight adult wing [28]. We tested if dKDM5/LID too can affect wing development. We utilized both of the above-mentioned UAS-RNAi lines crossed to the tissue-specific *Ser*-*GAL4* driver line to reduce expression of dKDM5/LID in wing imaginal disc cells. Knockdown of *lid* in wing discs using either RNAi line resulted in a curved wing phenotype similar to the phenotype observed upon *Sin3A* knockdown (Fig. 3a and Additional file 1: Figure S2A). Double knockdown of *Sin3A* and *lid* in wing discs resulted in a more severe curved wing phenotype than knockdown of either gene alone (Fig. 3a and Additional file 1: Figure S2A).

Use of a second wing imaginal disc-specific driver line, *Bx*-*GAL4*, also resulted in a curved wing phenotype upon knockdown of *lid* (data not shown). Interestingly, in addition to the curved wing phenotype, additional vein defects were observed upon wing-specific knock down of *lid* using the *Bx*-*GAL4* driver (Fig. 3b). The differences in expression patterns of *Serrate* (*Ser*) and *Beadex* (*Bx*) in wing imaginal discs may contribute to the additional vein defects observed when *lid* was knocked down using the *Bx*-*GAL4* driver. Wing vein disruptions have previously been observed in a *lid* mutant background upon additional mutation of *Suppressor of Hairless* (*Su(H)*), a regulator of Notch signaling [48]. Of note, the vein defects were only observed in male *lid* knockdown flies. One possible explanation for this observation is the fact that *Bx* is located on the X chromosome and thus can be subjected to regulation by the dosage compensation complex. Possible increased expression of the sequence targeting degradation of *lid,* due to dosage compensation, could thereby result in a more severe RNAi effect. Alternately, it is possible that male-specific developmental requirements for dKDM5/LID exist that can affect wing morphology. Published work suggests that male flies are more sensitive to mutations in *lid* compared to female siblings [40]. These researchers found that male flies carrying a demethylase inactive *lid* gene are short lived and display increased sensitivity to paraquat relative to female mutants. Similarly, RNAi-mediated knockdown of a chromatin regulatory protein MRG15, which associates with dKDM5/LID, also results in shortened lifespan, which is more prominent in male flies compared to females [49].

Apart from testing the effect of reduced dKDM5/LID levels, we further analyzed the role of dKDM5/LID in development through overexpression. Similar to the reduction of dKDM5/LID, ubiquitous overexpression of dKDM5/LID using the *Act*-*GAL4* driver line resulted in complete lethality when flies were reared at 27 °C (Table 1). This observation suggests that total levels of dKDM5/LID must be maintained; too much or too little is detrimental to fly development. Next, we overexpressed dKDM5/LID only in wing imaginal discs using the *Ser*-*GAL*4 driver. The resulting progeny had a held-out wing phenotype, sometimes with scalloped wing margins (Fig. 3c and Additional file 1: Figure S2B). Overexpression of a dKDM5/LID catalytic mutant resulted in more severe held-out wings with very distinctly scalloped wing margins. The held-out wing and scalloped wing margin phenotypes were more pronounced in male flies compared to females.

The more severe curved wing phenotype observed upon double knockdown of *Sin3A* and *lid*, the vein disruption phenotype observed upon *lid* knockdown and the held-out wing phenotype observed upon dKDM5/LID overexpression all imply that dKDM5/LID may have additional roles in wing development that are distinct from the SIN3 complex function. Taken together, our data indicate that dKDM5/LID is an essential factor for normal wing morphology. The results presented here are consistent with previous reports indicating that mutations in dKDM5/LID enhance or suppress wing venation defects caused by mutations of transcriptional regulators such as Notch, Hairless, Lsd1, and SNR1 [6, 50, 51]. In this work, we demonstrate that knockdown of *lid* by itself can cause wing morphological defects and that SIN3 and dKDM5/LID may function coordinately during wing development.

### Overexpression of dKDM5/LID partially rescues the *Sin3A* knockdown wing phenotype

Specific reduction of either SIN3 or dKDM5/LID in the wing imaginal disc resulted in a curved wing (Fig. 3a). We next wanted to test if overexpression of dKDM5/LID could rescue the wing defect caused by reduction of SIN3. Two fly lines previously generated in our laboratory, *SIN3 KD I* and *SIN3 KD II*, which have constitutive knock down of *Sin3A* in wing imaginal discs, were utilized for this analysis [28, 52]. *SIN3 KD I* and *SIN3 KD II* transgenic flies display a curved wing phenotype with 100 % penetrance. We crossed the *SIN3 KD I* or *SIN3 KD II* flies to flies carrying a UAS construct for overexpression of dKDM5/LID. To determine the amount of possible rescue, we scored for straight wings in the progeny. Here we have only considered the decrease of the penetrance of the curved wing phenotype and not suppression of the phenotype itself. That is, wings that were less curved were scored as curved and not straight. Approximately 95 % of the female progeny resulting from the cross had straight wings, indicating rescue of the curved wing phenotype (Table 2). Such rescue of the curved wing, however, was not observed in male flies. This finding further indicates the possibility that male-specific developmental requirements for SIN3 and or dKDM5/LID exist that can affect wing morphology. Male flies may require high levels of these proteins during wing development and thus may not be compensated by the level of overexpression achieved in our rescue experiments. The rescue of the wing phenotype in females suggests that increased expression of dKDM5/LID can compensate for the reduction of SIN3 function, possibly due to overlapping roles of these proteins in wing development. Further, unlike knockouts, RNAi does not often result in complete loss of the proteins. Therefore, the SIN3 protein could still be expressed in low levels. It is possible that overexpression of dKDM5/LID results in the sequestering and efficient utilization of the small amounts of SIN3 available, thereby resulting in wild-type wings. Overexpression of a catalytic mutant dKDM5/LID in the *SIN3 KD I* flies resulted in less than 5 % decrease in the penetrance of the curved wing in female flies and no rescue in the males. This lack of effect on female SIN3 KD flies suggests an important role for the demethylase activity of dKDM5/LID in the observed genetic interaction. Genome-wide analysis of histone modification patterns in *Drosophila* species have revealed variations in the chromatin landscape between the sexes [53]. It is possible that the histone demethylase activity of dKDM5/LID can contribute to such variation in chromatin structure between males and females, which in turn may explain the sex-specific requirements for dKDM5/LID observed in our genetic studies.

**Table 2:** Overexpression of dKDM5/LID rescues the *Sin3A* knockdown curved wing phenotype in female flies

Genotype	♂		♀	
	% Curly	Flies scored	% Curly	Flies scored
*SIN3 KD I*	100		100	
*SIN3 KD II*	100		100	
*SIN3 KD I/EGFP* ^(2)^	100	50	100	92
*SIN3 KD I/LID* ^(3)^	100	134	4.5 ± 7.7	303
*SIN3 KD II/LID* ^(2)^	100	122	3.8 ± 5.4	132
*SIN3 KD I/LID*-JmjC* ^(2)^	100	100	97.5 ± 2.2	175

Flies constitutively knocked down for *Sin3A* (SIN3 KD) in wing imaginal discs were crossed to UAS overexpression fly lines for EGFP (control), *lid* or mutant *lid* and the progeny counted and analyzed for curved wings

^(n)^ Number of trials

*KD* knockdown

### SIN3 and dKDM5/LID affect transcription of common and distinct genes

Having determined that both SIN3 and dKDM5/LID affect cell proliferation and wing development in flies, we wished to determine the possible underlying transcriptional network regulated by these proteins. Therefore, we utilized the highly sensitive RNAseq approach to identify genome-wide changes in gene expression upon RNAi-mediated reduction of SIN3 or dKDM5/LID or both in *Drosophila* S2 cells. S2 cells treated with dsRNA targeting *GFP* was utilized as a control. Three biological replicates of the RNAseq data were generated and Pearson’s correlation analysis indicated strong reproducibility of the data (Additional file 1: Figure S3).

**Fig. 4:**
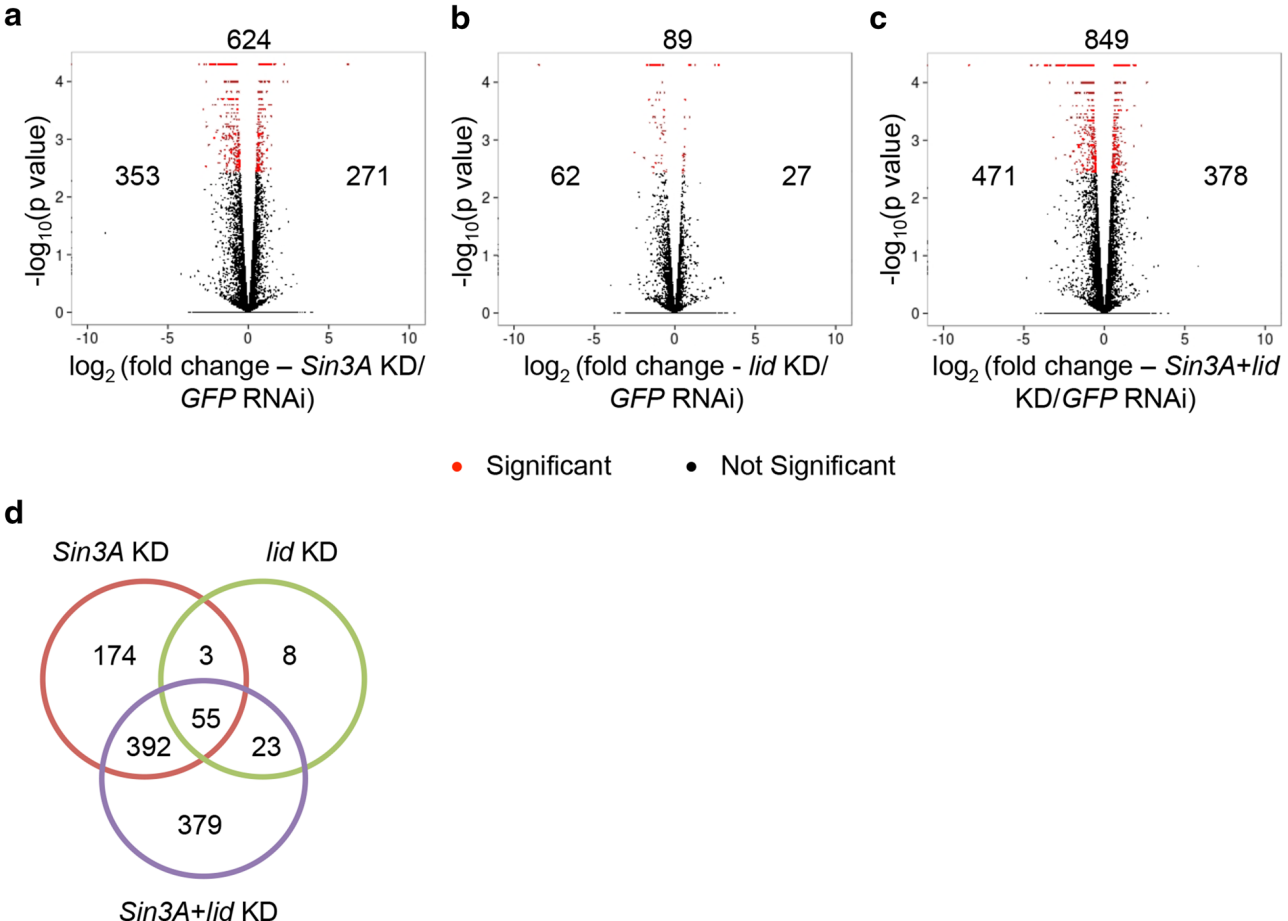
Genes regulated by SIN3, dKDM5/LID, or both in S2 cells as determined by RNAseq. **a**–**c** Volcano plots depicting log fold change in expression of indicated samples. The total number of genes with significant changes in expression is displayed above the plot, while the number of upregulated genes is indicated to the *right* and downregulated genes to the *left*. **d** *Venn diagram* indicating overlap of genes regulated by SIN3 and dKDM5/LID. *KD* knockdown

Differential expression analysis was performed comparing knockdown samples to control. Genes showing expression changes greater than 1.4-fold with an FDR cutoff of 0.05 were identified as significantly regulated targets (Fig. 4 and Additional file 2). 624 and 89 genes were determined as regulated by SIN3 and dKDM5/LID, respectively. Dual knockdown of *Sin3A* and *lid* resulted in the misregulation of 849 genes. In addition, a general increase in the amount of expression change was also seen at most genes upon dual knockdown compared to the single knockdowns (Additional file 1: Figure S4 and Additional file 2). The increase in the number of regulated genes as well as the increase in the amount of expression changes, observed upon dual knockdown of *Sin3A* and *lid* compared to single knockdown, suggests an additive role for these proteins in regulating transcription of many gene targets. This work has identified a significant number of novel genes regulated by SIN3 or dKDM5/LID in *Drosophila* S2 cells.

Of the 89 genes regulated by dKDM5/LID, a majority of genes (58, 65 %) overlapped with SIN3-regulated genes (Fig. 4d). This result supports the idea that dKDM5/LID could function coordinately with SIN3 as part of a complex to regulate gene transcription and downstream processes. However, the genes that were regulated by only SIN3 or dKDM5/LID, indicate unique transcription regulatory roles for these proteins in addition to a common gene regulatory role as part of the SIN3 complex.

Of the genes misregulated upon knockdown of *Sin3A*, 271 (43 %) were upregulated and 353 (57 %) genes were downregulated (Fig. 4a). This somewhat equal distribution of genes that were upregulated or downregulated is in contrast to previous work indicating that SIN3 functions predominantly as a repressor [16]. The previously published microarray data used a combination of gene expression data obtained from both S2 and Kc cell lines. It is possible that activation of genes by SIN3 is more tissue and cell type specific compared to repression of target genes. Knockdown of *lid*, however, resulted in twice as many genes to be downregulated, where 27 (30 %) genes were upregulated and 62 (70 %) were downregulated (Fig. 4b). This corroborates the published microarray expression data from *Drosophila* wing imaginal disc tissue establishing a predominant role for dKDM5/LID in gene activation [38, 39]. Double knockdown of *Sin3A* and *lid* show a similar trend to *Sin3A* knockdown, where upregulated genes (378, 45 %) were relatively equal in number to downregulated genes (471, 55 %) (Fig. 4c).

To verify the RNAseq data we repeated the RNAi knockdown experiments and analyzed isolated RNA by real-time qRT-PCR. For this purpose, we tested 12 candidate genes (Additional file 1: Figure S5A–C). Real-time qRT-PCR results validated the expression trends observed by RNAseq.

**Fig. 5:**
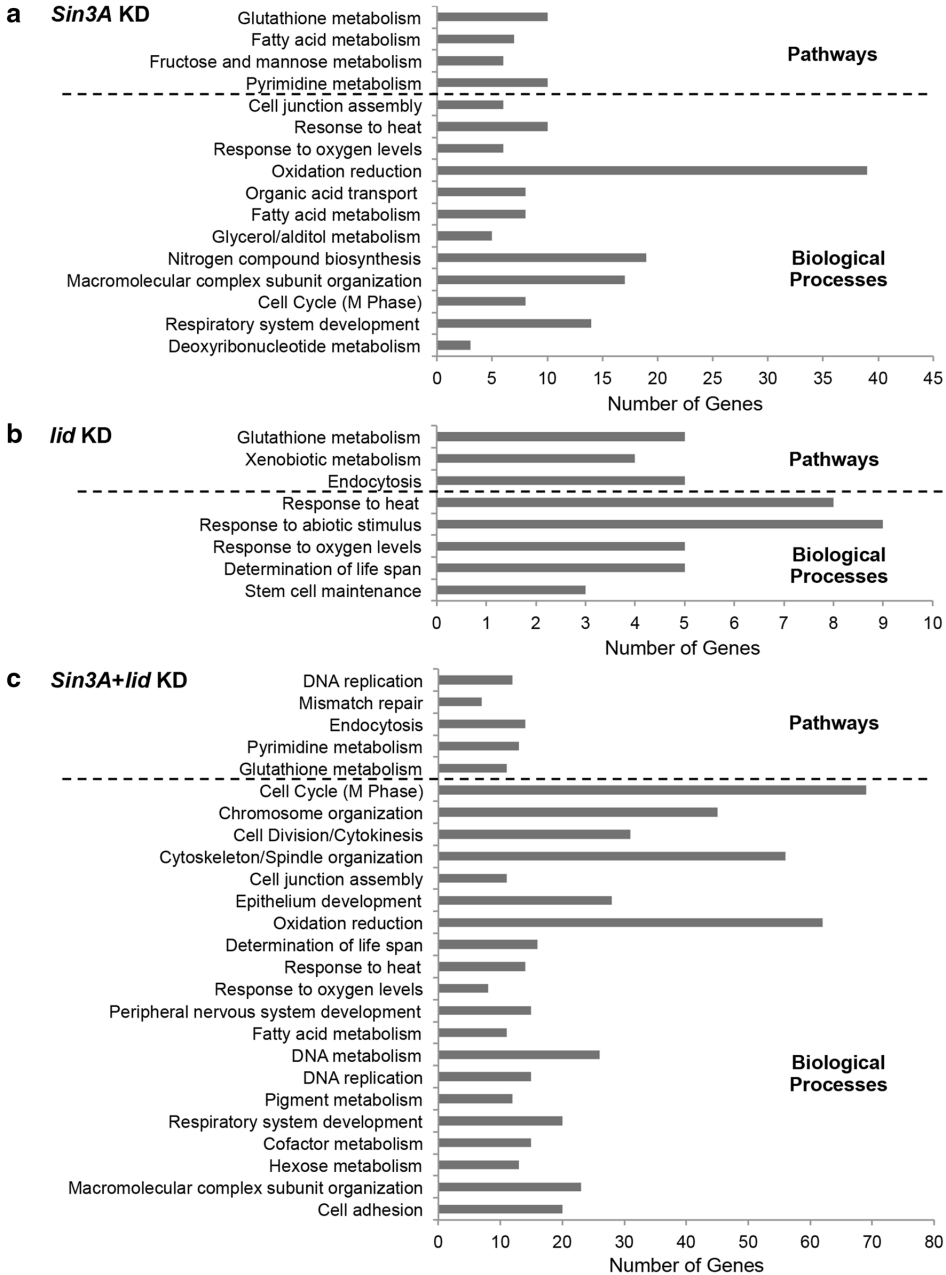
Genes regulated by SIN3, dKDM5/LID, or both are enriched in similar and distinct biological processes **a**–**c** Gene ontology (GO) and pathway analysis of genes regulated by SIN3, dKDM5/LID, or both as indicated. Related GO terms with considerable overlap of genes have been combined. *P*  < 0.01. *KD* knockdown

We next used the database for annotation, visualization and integrated discovery (DAVID) and conducted Gene ontology (GO) and KEGG pathway analysis of genes identified by RNAseq [54]. SIN3-regulated genes were classified into overrepresented processes including cell junction assembly, stress response, metabolic, cell cycle, and developmental processes (Fig. 5a and Additional file 3). Stress-associated processes such as heat shock response, lifespan determination, and glutathione metabolism were significantly enriched among dKDM5/LID-regulated genes (Fig. 5b and Additional file 3). Simultaneous knockdown of *Sin3A* and *lid* resulted in expression changes of genes involved in similar processes to those in the single knockdowns, such as cell cycle, metabolism, and stress tolerance (Fig. 5c and Additional file 3). Comparatively higher overrepresentation of cell cycle genes was observed upon dual knockdown relative to the single knockdowns.

As individual knockdown of *Sin3A* and *lid* both affect cell proliferation (Fig. 2), genes involved in cell cycle-related processes that are regulated by SIN3 and dKDM5/LID are of specific interest. In *Drosophila*, RNAi-mediated knockdown of *Sin3A* leads to cell cycle arrest at the G2/M phase [17]. In mammals, KDM5 proteins have been linked to cell cycle regulation through the E2F/RB pathway, which affects G1/S transition [55]. Our gene expression analysis determined that *Sin3A* knockdown led to expression changes in several genes involved in mitotic spindle organization including downregulation of the mitotic kinase *polo*, the Cyclin-dependent kinase *Cdk4*, and the inhibitory checkpoint kinase *grapes* (*grp*). While *lid* knockdown did not result in significant changes in cell cycle genes, the double knockdown of *Sin3A* and *lid* resulted in expression changes of many additional cell cycle genes including the Cyclin-dependent kinase *Cdk1*/*Cdc2*. The data suggest that the additive effect due to the simultaneous reduction of both SIN3 and dKDM5/LID is important to bring about significant changes in the expression of many cell cycle genes. Knockdown of *lid*, however, resulted in phenotypic changes in cell proliferation (Fig. 2). Thus it is possible that the small changes in expression observed at cell cycle genes upon *lid* knockdown, while not statistically significant, are biologically relevant.

Many cell cycle regulators are also known to affect wing development [56]. Apart from cell cycle regulatory genes, many genes implicated in wing development were also affected by SIN3 and/or dKDM5/LID. Genes such as *Notch* (*N*), *Ser*, and *Daughters against dpp* (*Dad*) were affected by knockdown of either *Sin3A* or *lid*. *Thor* and *Sequence*-*specific single*-*stranded DNA*-*binding protein* (*Ssdp*) were regulated by SIN3 alone or dKDM5/LID alone, respectively. Genes such as *suppressor of Hairy wing* (*su(Hw)*) were affected only in the double knockdown condition. Wing imaginal discs have previously been utilized to show the interconnection of pathways involved in growth, proliferation, and developmental patterning [57]. Thus effects on genes implicated in both cell cycle and wing development could lead to the observed wing developmental defects (Fig. 3).

Cell junction assembly was among the enriched processes involving genes regulated by SIN3. Genes such as *Neurexin IV* (*Nrx*-*IV*), *Contactin* (*Cont*), *sinuous* (*sinu*), and *Gliotactin* (*Gli*) involved in cell junction assembly are implicated in the maintenance of blood–brain barrier and heart morphogenesis [58–60]. Interestingly, genetic screens have previously identified SIN3 as a regulator of cardiac and neural development [25–27]. Identification of the above septate junction assembly genes as SIN3 targets could aid future work in determining how SIN3 regulates these developmental processes.

A large number of SIN3-regulated genes were involved in multiple metabolic processes. Double knockdown of *Sin3A* and *lid* resulted in the misregulation of a larger number of genes implicated in metabolic pathways compared to single knockdown of *Sin3A*. The enrichment of genes involved in metabolic processes is consistent with published work from our laboratory [16, 61]. We previously determined that SIN3 regulates many genes involved in cytosolic and mitochondrial metabolic pathways that control cellular energy production. The current data add to the previous work identifying novel genes involved in multiple metabolic processes implicating an important role for SIN3 in metabolic homeostasis.

A highly enriched category of genes regulated by both SIN3 and dKDM5/LID are genes involved in stress tolerance mechanisms (Fig. 5). A majority of these genes are heat shock response genes. Heat shock proteins are activated in response to multiple cellular stresses such as heat, oxidative stress, toxins, and bacterial infections and help counteract proteotoxicity and thereby influence organismal lifespan [62, 63]. Some heat shock proteins are also induced by the JNK signaling pathway and the transcription factor Foxo, which are also implicated in lifespan determination [64]. SIN3 and dKDM5/LID have each been associated with lifespan determination [19, 38, 40, 65]. Interestingly, an RNAi screen genetically links SIN3 to the JNK signaling pathway, where SIN3 acts as an enhancer of JNK phosphorylation [24]. Recently, dKDM5/LID was reported to interact with Foxo and help recruit Foxo to genes coregulated by dKDM5/LID and Foxo [38]. Understanding the coordinate function of these stress response regulators is anticipated to provide further insight to the mechanisms of lifespan extension.

Another set of genes regulated by both SIN3 and dKDM5/LID are the Glutathione S transferase genes, involved in glutathione metabolism. Glutathione is an antioxidant, which plays a key role in the defense against oxidative stress and is also implicated in the modulation of cell proliferation and cell death [66]. In *Drosophila*, glutathione supplementation results in increased survival of flies treated with the oxidative stress paraquat [19, 67]. Thus, the glutathione pathway may be a critical link to the observed phenotypes associating SIN3 and dKDM5/LID to stress tolerance, lifespan extension, and cell proliferation.

### Oxidative stress augments gene expression changes due to reduced SIN3 and dKDM5/LID

The RNAseq data indicated that both SIN3 and dKDM5/LID regulated several genes that are implicated in stress tolerance processes. To identify genes regulated by SIN3 and dKDM5/LID under conditions of oxidative stress, we treated cells with paraquat. Paraquat treatment generates the reactive oxygen species (ROS), superoxide anion, where accumulation of ROS results in oxidative stress in the cell [68]. S2 cells knocked down for *Sin3A*, *lid*, or both or treated with dsRNA against *GFP* were further subjected to paraquat treatment and analyzed by RNAseq. S2 cells treated with dsRNA against *GFP* but not treated with paraquat were used as the control.

**Fig. 6:**
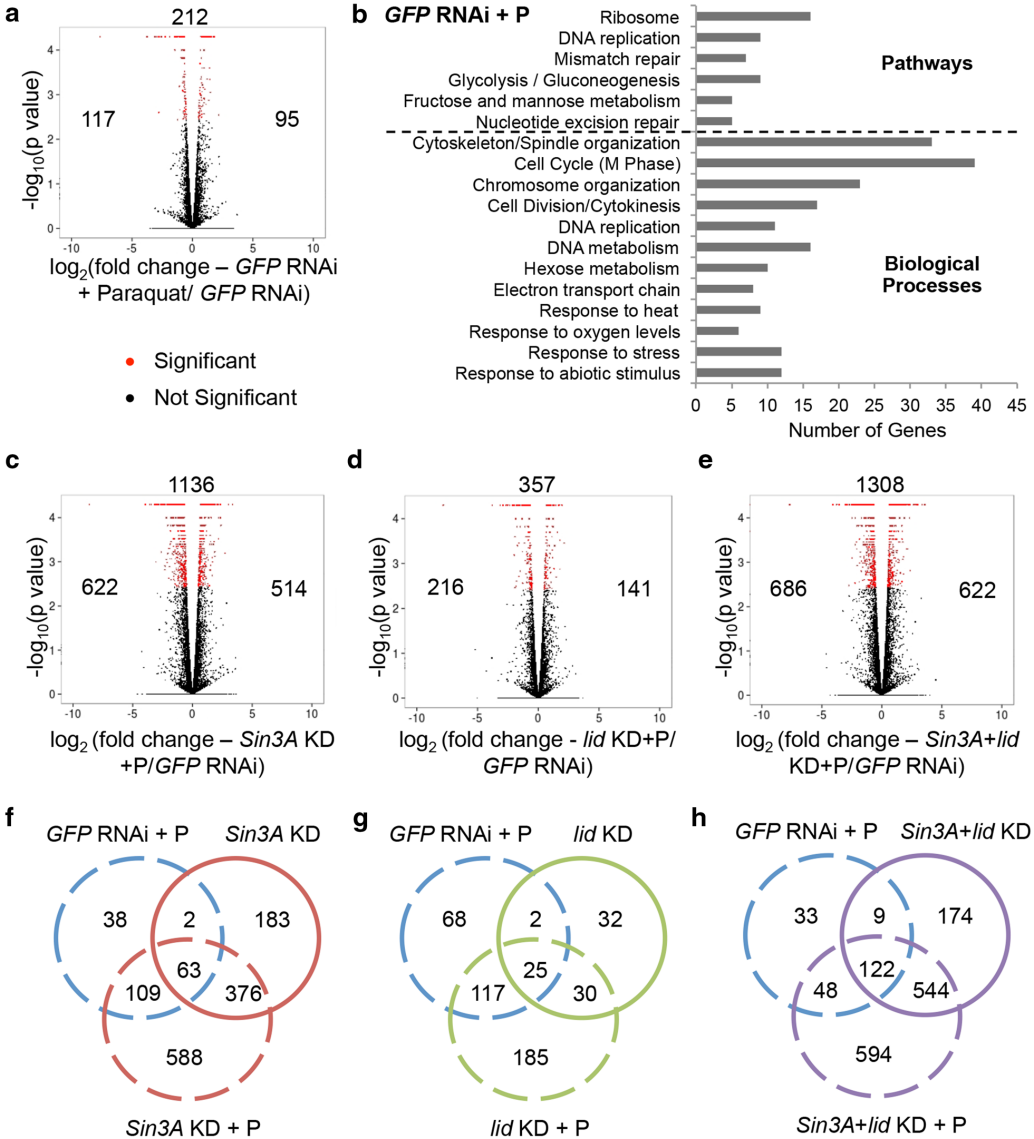
Genes regulated by SIN3, dKDM5/LID, or both under the induction of paraquat-mediated oxidative stress in S2 cells as determined by RNAseq. (**a**, **c**–**e**) Volcano plots depicting log fold change in expression. The total number of genes with significant changes in expression is displayed *above* the plot, while the number of upregulated genes is indicated to the *right* and downregulated genes to the *left*. **b** Gene ontology (GO) and pathway analysis of genes that change in expression upon induction of oxidative stress. Related GO terms with considerable overlap of genes have been combined. *P* < 0.01. **f**–**h** *Venn diagrams* indicating overlap of genes regulated by SIN3 and dKDM5/LID under normal and stress-induced conditions. *KD* knockdown, *P* paraquat

As above, genes with a fold change greater than 1.4 and an FDR cutoff of 0.05 were considered as significantly regulated targets. Induction of oxidative stress in *GFP* dsRNA-treated cells resulted in the expression changes of 212 genes relative to the non-stressed *GFP* RNAi control (Fig. 6a). GO and KEGG pathway analysis indicated that genes that responded to paraquat-mediated oxidative stress fell into a number of overrepresented categories including cell cycle, DNA replication and repair, metabolism, and stress tolerance (Fig. 6b).

Under oxidative stress conditions, knockdown of *Sin3A*, *lid,* or both resulted in gene expression changes in a larger number of genes compared to non-stressed conditions (Figs. 4a–c and 6c–e). 588, 185, and 594 genes were affected upon knockdown of *Sin3A*, *lid*, or both during paraquat treatment, but did not significantly change in similar knockdown during non-stressed conditions or upon induction of stress alone (Fig. 6f–h). Comparing stressed to non-stressed conditions, in addition to a larger number of genes affected in the stress condition, an increase in the amount of expression change was also noted at many genes (Additional file 1: Figure S4 and Additional file 2). Expression changes observed by RNAseq analysis were validated by real-time qRT-PCR analysis of selected genes (Additional file 1: Figure S5D–G).

**Fig. 7:**
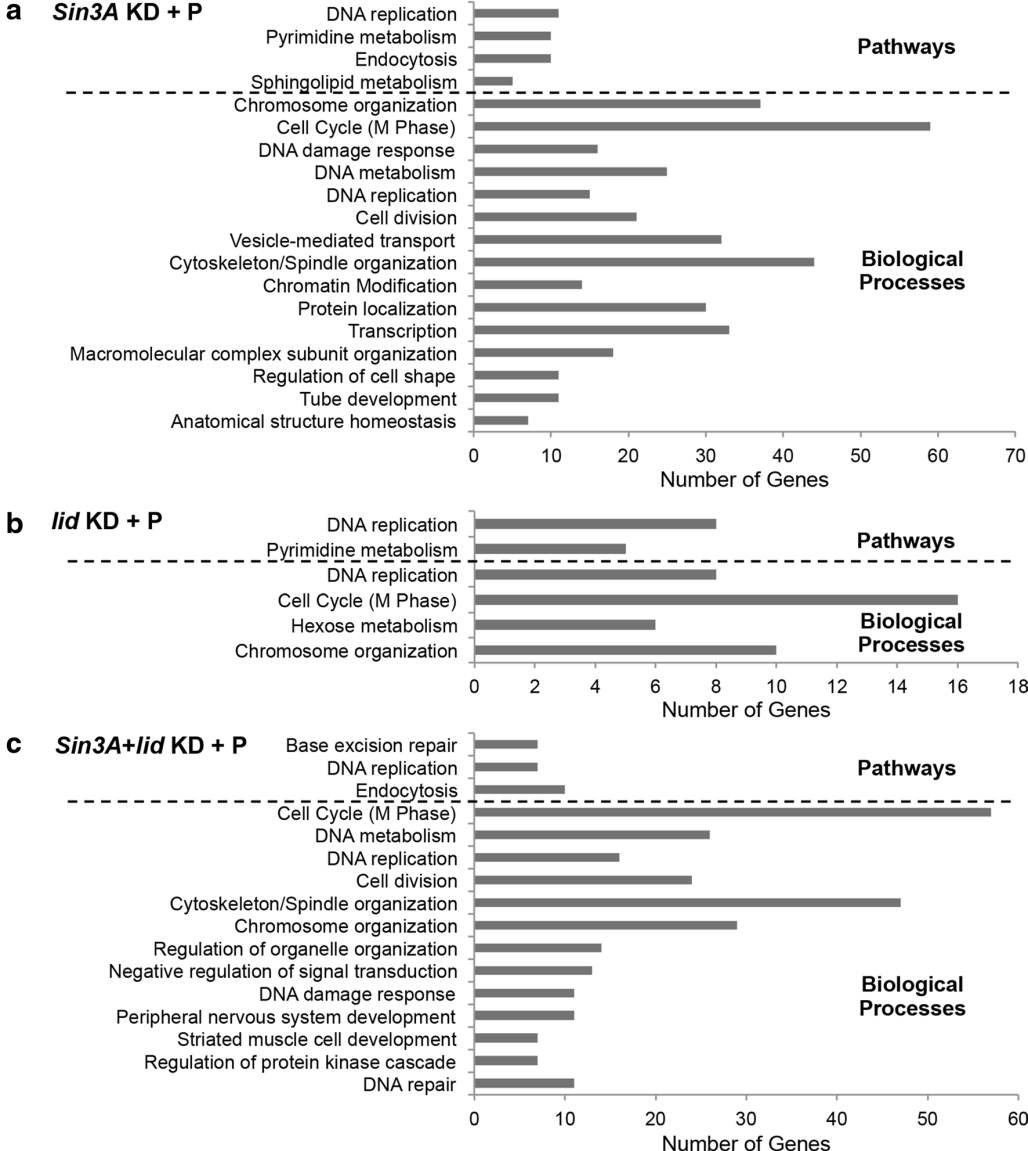
Genes regulated by SIN3 and/or dKDM5/LID under oxidative stress conditions are enriched in cell cycle-related processes. **a**–**c** Gene ontology (GO) and pathway analysis of additional genes that change in expression upon knockdown of *Sin3A*, *lid*, or both upon induction of oxidative stress that do not significantly alter expression under normal conditions or upon induction of stress alone. Related GO terms with considerable overlap of genes have been combined. *P*  < 0.01. *KD* knockdown, *P* paraquat

As we wished to discover the impact of SIN3 and dKDM5/LID during oxidative stress, we performed GO and pathway analysis of those genes affected upon knockdown of *Sin3A*, *lid*, or both in oxidative stress conditions but whose expression was unchanged in the RNAi cells in normal conditions or in cells subjected solely to stress. Interestingly, cell cycle and DNA replication-related processes were the most enriched under all conditions (Fig. 7a–c). This result was different from that obtained in non-stressed conditions, where cell cycle-related processes were most enriched only upon double knockdown of *Sin3A* and *lid*. The process of cell cycle was also enriched in the comparison of genes affected by paraquat-mediated oxidative stress to control cells (Fig. 6b). These results indicate that the effect on cell cycle-related processes caused by an environment of oxidative stress is exacerbated when combined with the reduction of SIN3 or dKDM5/LID proteins. *Cyclin A* and *B* (*CycA* and *CycB*) and the phosphatase *String* (*Stg*), implicated in the G2/M transition, as well as *Cyclin E* (*CycE*), the Cyclin-dependent kinase *Cdk2*/*Cdc2c* and the inhibitor *dacapo* (*dap*), involved in G1/S transition of the cell cycle, were among the genes affected upon the loss of *Sin3A*, *lid*, or both under stress conditions. Interestingly, the majority of the cell cycle target genes were downregulated upon reduction of these proteins. This result indicates that SIN3 and dKDM5/LID are important for activation, rather than repression, of those particular targets. Surprisingly, both activators and inhibitors of cell cycle progression are downregulated upon knockdown of *Sin3A* and or *lid*. These data suggest that the cell cycle circuitry is misregulated under these varied stress conditions. To test this prediction, we measured cell cycle progression of S2 cells and cells with knockdown of *Sin3A*, *lid*, or both under paraquat-mediated stress conditions by flow cytometry. In accord with the RNAseq analysis, induction of paraquat-mediated oxidative stress resulted in a G2/M phase arrest or delay (Additional file 1: Figure S6). Knockdown of *Sin3A*, *lid*, or both under oxidative stress conditions exacerbated this phenotype. Further, the flow cytometry analysis reveals a qualitatively stronger defect in cell cycle progression in the oxidative stress conditions compared to normal conditions upon reduction of *Sin3A*, *lid*, or both. This is consistent with the RNAseq analysis, where an increased number of genes involved in cell cycle regulation were significantly misregulated under oxidative stress conditions relative to control conditions.

### Reduction of SIN3 or dKDM5/LID partially mimics oxidative stress in cells

Our gene expression data further led to the interesting observation that many genes regulated by SIN3 or dKDM5/LID under normal conditions were also misexpressed upon induction of oxidative stress in cells treated with dsRNA to *GFP*. 65 genes regulated by SIN3 (Fig. 6e) and 27 genes regulated by dKDM5/LID (Fig. 6f) were also misregulated upon paraquat treatment of control cells. 131 genes were misregulated upon double knockdown of *Sin3A* and *lid* and upon paraquat treatment of control cells (Fig. 6g). Thus, there is considerable overlap between the genes involved in a response to oxidative stress and those regulated by SIN3 and/or dKDM5/LID in non-oxidative stress conditions. This set includes the heat shock response genes, which were misregulated in all knockdown conditions and in control cells treated with paraquat. As expected, based on the overlap in genes, GO analysis revealed that similar processes, including stress tolerance processes, were overrepresented upon paraquat treatment of control cells and knockdown of *Sin3A*, *lid*, or both under normal conditions (Figs. 5a–c and 6b). Our results implicate SIN3 and dKDM5/LID in playing a critical role in stress tolerance and suggest that the loss of these proteins may partially mimic oxidative stress conditions in the cell.

### SIN3 and dKDM5/LID bind to the TSS proximal regions of target genes

Having established that SIN3 and dKDM5/LID can alter expression levels of common and unique gene targets, we wanted to see if SIN3 and dKDM5/LID directly bound these genes. We analyzed binding of SIN3 and dKDM5/LID at several genes identified by RNAseq as being regulated by SIN3, dKDM5/LID, or both under normal or oxidative stress conditions using chromatin immunoprecipitation-quantitative PCR (ChIP-qPCR). Flybase GO terms were also taken into consideration in the selection of genes for testing [69]. We tested *varicose* (*vari*), a gene involved in cell junction assembly, a process highly enriched among SIN3-regulated genes [70]. Metabolic genes were highly enriched among SIN3-regulated genes. Therefore we included *Cytochrome c proximal* (*Cyt*-*c*-*p*), *CG3476, mitochondrial ribosomal protein L19* (*mRpL19*), *Glutathione S transferase E6* (*GstE6*), and *S*-*adenosylmethionine synthetase* (*Sam*-*S*), genes involved in multiple metabolic processes [71–75]. Due to the roles of both SIN3 and dKDM5/LID in cell proliferation and wing development, we tested several genes implicated in these processes. *Minichromosome maintenance 7* (*Mcm7*), *Thor, and Sestrin* (*Sesn*) were chosen for their involvement in cell cycle functions. [76–81]. *Thor and Sesn* have also been implicated in wing development due to their roles in regulating cell size [78, 79]. We further tested *Ssdp*, *Heat shock protein 27* (*Hsp27*), and *interference hedgehog* (*ihog*), all implicated in wing development [82–85]. *Thor*, *Sesn*, and *Hsp27* are also involved in stress response and lifespan determination pathways [78, 86–88]. *CG31819*, a gene located in a region devoid of SIN3 or dKDM5/LID binding based on published genome-wide binding data, was used as a negative control [39, 89, 90].

*Drosophila* S2 cells predominantly express SIN3 220, the largest isoform. We utilized *Drosophila* S2 cells that express either HA-tagged SIN3 220 or FLAG-HA-tagged dKDM5/LID. S2 cells not carrying any transgene were used as a control. We prepared chromatin from control cells or cells expressing the HA-tagged proteins and immunoprecipitated using anti-HA beads. Real-time qPCR was used to determine enrichment of SIN3 and dKDM5/LID at the selected gene targets. We designed qPCR primers that amplify regions spanning the TSS of selected genes based on published genome-wide binding studies for SIN3 and dKDM5/LID [39, 89, 90].

**Fig. 8:**
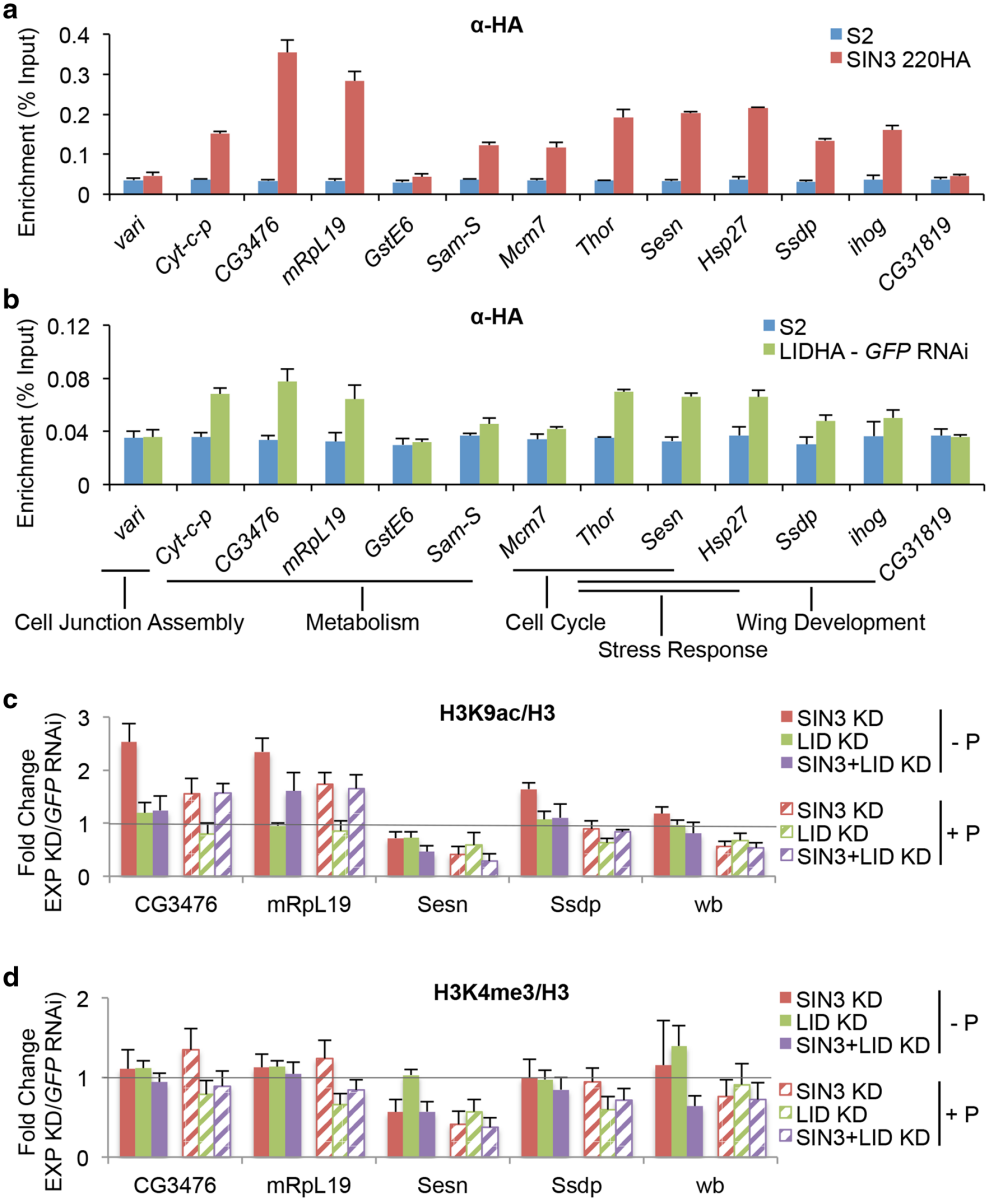
SIN3 and dKDM5/LID bind to common gene targets. Real-time quantitative PCR analysis of chromatin prepared from indicated cell lines immunoprecipitated with beads conjugated to antibody to the HA tag or antibody to SIN3. **a** Enrichment of SIN3 220 at predicted target genes. **b** Enrichment of dKDM5/LID at target genes. Primers used in the PCR amplification of target regions spanning the TSS of indicated genes. *CG31819* acts as a negative control. The results are the average of three biological replicates. **c** Enrichment of H3K9ac at promoter proximal regions of SIN3 and/or dKDM5/LID target genes upon knockdown of *Sin3A*, *lid*, or both under normal and oxidative stress conditions. **d** Enrichment of H3K4me3 at promoter proximal regions of SIN3 and/or dKDM5/LID target genes upon knockdown of *Sin3A*, *lid*, or both under normal and oxidative stress conditions. *KD* knockdown, *P* paraquat

Substantial enrichment of SIN3 220 compared to control at most of the tested genes, with the exception of *vari* and *GstE6*, was observed (Fig. 8a). No enrichment was noted at the negative control gene *CG31819*. We further validated binding of SIN3 to target genes by immunoprecipitating SIN3 from S2 cells using an antibody targeting endogenous SIN3 protein. Chromatin from S2 cells was also immunoprecipitated with IgG as a control. Enrichment at gene targets was observed for both endogenous SIN3 protein and HA-tagged SIN3 protein (Fig. 8a and Additional file 1: Figure S7A). No significant enrichment was observed in the control IP (Additional file 1: Figure S7A). Further, knockdown of *Sin3A* by RNAi led to decreased binding of SIN3, verifying direct binding of SIN3 at tested gene targets (Additional file 1: Figure S7B).

Of the two tested genes having no significant enrichment of SIN3 at the TSS, *vari* expression was upregulated upon depletion of *Sin3A* under both normal and oxidative stress conditions. Thus, the binding data suggests that this gene may be an indirect target of SIN3. Upregulation of *GstE6* expression by SIN3 was only observed under paraquat-mediated oxidative stress conditions. Thus it is possible that SIN3 is recruited to this gene only under such conditions. Of the genes with significant enrichment of SIN3, *CG3476*, *mRpL19*, *Thor*, and *Sam*-*S* are negatively regulated by SIN3. *Sesn*, *Hsp27*, and *Mcm7* are positively regulated by SIN3. *Cyt*-*c*-*p* and *ihog* were only significantly upregulated upon knockdown of *Sin3A* under induction of oxidative stress. Knockdown of *Sin3A* did not result in any significant changes in the expression of *Ssdp*, yet the TSS of this gene was bound by SIN3. The varying transcriptional outcomes of genes bound by SIN3 suggest a complex transcriptional program involving SIN3. The histone-modifying functions of SIN3-associated proteins RPD3 and dKDM5/LID remove marks associated with active transcription suggesting a role in gene repression. The binding data indicates that SIN3 can directly activate or repress gene targets, implying distinct mechanisms of gene regulation by the SIN3 complex.

Compared to SIN3, dKDM5/LID was only modestly enriched at tested genes, where all genes showed less than 2.5-fold enrichment compared to control (Fig. 8b). Only *Cyt*-*c*-*p*, *CG3476*, *Thor*, *Sesn*, *Hsp27*, and *ihog* had considerable enrichment compared to control. All genes bound by SIN3, however, showed above background levels of enrichment for dKDM5/LID. These observations suggest that both SIN3 and dKDM5/LID may be recruited together as a complex to gene targets. The low enrichment of dKDM5/LID may arise due to a lower stoichiometry of the protein at gene targets compared to SIN3 or due to varying DNA:protein crosslinking efficiencies for SIN3 and dKDM5/LID. Of the genes bound by dKDM5/LID, significant transcriptional regulation was seen only for *Sesn* and *Hsp27*, where both genes are downregulated upon *lid* knockdown. *Cyt*-*c*-*p* and *CG3476* are upregulated upon *lid* knockdown under conditions of oxidative stress. All genes with dKDM5/LID enrichment were also enriched for SIN3. It is likely that due to interaction with the SIN3 complex, dKDM5/LID too binds to gene targets bound by SIN3, while its effect on transcriptional regulation is restricted to a subset of these genes. Common and unique protein interactions of SIN3 and dKDM5/LID may contribute to the similar and distinct transcriptional outcomes at different gene targets.

To further understand transcriptional regulation by SIN3 and dKDM5/LID, we wished to analyze possible underlying changes in histone modifications at target genes upon reduction of SIN3, dKDM5/LID, or both. We studied histone H3K9ac, a mark that can be deacetylated by the SIN3 complex protein, RPD3, and H3K4me3, a mark that can be removed by dKDM5/LID [17, 30–33]. For this purpose, we isolated chromatin from RNAi-treated S2 cells under normal conditions as well as paraquat-induced oxidative stress conditions. Chromatin was subjected to immunoprecipitation with antibody against H3K9ac and H3K4me3. Immunoprecipitation with antibody to the H3 C-terminus was used to normalize for changes in histone density. IgG was used as a control for immunoprecipitation. ChIP-qPCR was performed as above to investigate possible changes in histone modification patterns.

We monitored the enrichment of histone modification marks at four genes bound by SIN3 and dKDM5/LID, namely *CG3476*, *mRpL19*, *Sesn*, and *Ssdp*. *CG3476* and *mRpL19* are genes that are repressed by SIN3, while *Sesn* is a gene that is activated by both SIN3 and dKDM5/LID and *Ssdp* by dKDM5/LID alone. Primers were designed to detect promoter proximal regions enriched for H3K9ac and H3K4me3 in S2 cells based on published data [91, 92]. Primers designed for the promoter region of *wb*, a gene not targeted by SIN3 or dKDM5/LID was used as a control.

Reduction of SIN3 led to an increase in H3K9ac at *CG3476* and *mRpL19,* genes upregulated upon knockdown of *Sin3A* under normal and oxidative stress conditions (Fig. 8c). Interestingly, while double knockdown of *Sin3A* and *lid* resulted in similar expression changes as single knockdown of *Sin3A*, no significant increase in acetylation was observed upon double knockdown under normal conditions (Fig. 8c and Additional file 1: Figure S5). Under oxidative stress conditions both single knockdown of *Sin3A* and double knockdown of *Sin3A* and *lid* resulted in an increase of H3K9ac at *CG3476* and *mRpL19*. In contrast, acetylation levels decreased at *Sesn,* a gene activated by both SIN3 and dKDM5/LID, upon knockdown of *Sin3A*, *lid,* or both under normal and oxidative stress conditions. It is of interest that reduced dKDM5/LID levels result in altered H3K9ac at *Sesn* as dKDM5/LID has no direct catalytic activity towards this histone modification. One previous report demonstrated that the in vitro HDAC activity of purified RPD3 could be inhibited by the addition of dKDM5/LID into the reaction [37]. It is possible that at genes activated by SIN3, dKDM5/LID inhibits the activity of RPD3 leading to transcriptional activation. Loss of either SIN3 or dKDM5/LID may disrupt the complex and thereby release the inhibition of RPD3 activity resulting in the observed changes in histone acetylation. Only moderate changes were noted at *Ssdp*, which may be attributed to the moderate transcriptional regulation of this gene by dKDM5/LID but not SIN3.

Little change in H3K4me3 was observed upon knockdown of *Sin3A*, *lid*, or both (Fig. 8d). While loss of *lid* is known to affect global levels of histone H3K4me3 [30–33], very little effect was seen near the TSS of the tested genes upon *lid* knockdown. A moderate decrease in H3K4me3, however, was noted upon *lid* knockdown or double knockdown of *Sin3A* and *lid* at most tested genes under conditions of oxidative stress. Unlike histone acetylation, histone methylation trends did not correlate with the gene expression trends for these tested genes. It is possible that histone methylation changes due to the catalytic activity of dKDM5/LID are context specific and help fine tune gene expression changes. Further, an important role for dKDM5/LID in the SIN3 complex may be as a modulator of deacetylase activity by RPD3, consistent with the study indicating that dKDM5/LID can inhibit RPD3 activity in vitro [37]. Overall the ChIP-qPCR results suggest affects on histone acetylation play a more predominant role relative to histone methylation in transcriptional regulation by the SIN3 complex.

## Conclusions

In conclusion, in this study, we have addressed the question as to whether dKDM5/LID affects similar processes as SIN3. We verified that dKDM5/LID interacts with SIN3 and the complex component, RPD3. We found that both SIN3 and dKDM5/LID share similar roles in cell proliferation and wing development, as flies with reduced dKDM5/LID phenocopied those with a reduction of SIN3. Through genome-wide expression and local chromatin recruitment analyses we determined that both SIN3 and dKDM5/LID bind to and regulate many common gene targets. A specific effect of SIN3 and dKDM5/LID was observed in the regulation of stress tolerance pathways, which in turn may affect cell cycle progression and wing development. Our findings therefore, provide a solid framework for analyzing the transcriptional network through which SIN3 and dKDM5/LID affect diverse functions in the cell. Further, our findings imply that dKDM5/LID is a key interactor of the SIN3 complex, where dKDM5/LID contributes significantly to SIN3 complex function in cellular and developmental processes. Moreover, in mammals, genes encoding SIN3 and KDM5 proteins have been linked to tumorigenesis [93, 94]. Understanding the coordinated functions of these proteins in cell cycle regulation could provide great insight for therapeutic development. While the SIN3 complex is best known for its association with the HDAC RPD3, the role of other components of the complex in modulating transcriptional control is less understood. Findings from this current work emphasize the importance of further understanding the contribution of dKDM5/LID to the activity of the SIN3 complex.

## Methods

### Cell culture

*Drosophila* Schneider cell line 2 (S2) cells (*Drosophila* Genomics Resource Center) were cultured at 27 °C in Schneider’s *Drosophila* medium (1×) + l-glutamine (Life Technologies) with 10 % heat-inactivated fetal bovine serum (Invitrogen) and 50 mg/ml gentamicin. 0.1 mg/ml penicillin/streptomycin and 0.1 mg/ml Geneticin for selection were added to cells carrying a stably integrated transgene with HA-tagged SIN3 187 or SIN3 220. Construction of the HA-tagged SIN3 187 and SIN3 220 expression cell lines has been previously described [7]. The FLAG-HA-tagged dKDM5/LID expressing cell line was generated by transfecting a pMK33 vector carrying FLAG-HA-tagged *lid* cDNA using the Effectene transfection kit (Qiagen). 300 μg/ml Hygromycin B was added to select for transfected cells carrying stable chromosomal insertions of the transgene. The FLAG-HA-tagged *lid* construct inserted into a pMK33 vector (FMO08240) was obtained from the Berkeley *Drosophila* Genome Project, ORFeome collection [95]. Expression of tagged proteins was induced by the addition of 1 μl/ml of 0.7 M CuSO_4_ to relevant cultured cells.

### Nuclear extract preparation and co-immunoprecipitation

Nuclear extracts were prepared from both S2 control and dKDM5/LID FLAG-HA stably transformed cells and subjected to immunoprecipitation as previously described [7]. In brief, approximately 900 μl of nuclear extract was incubated with 75 μl of anti-HA beads (monoclonal anti-HA agarose conjugate clone HA-7 (A2095, Sigma)). 150 μl of interaction buffer (20 mM HEPES (pH 7.4), 150 mM NaCl, 0.5 mM EDTA, 1 % Triton X-100, 10 % glycerol) was added to the extract and incubated with the antibody beads overnight at 4 °C. The beads were washed with radioimmune precipitation buffer (RIPA (20 mM Tris (pH 7.4), 150 mM NaCl, 1 % Triton X-100, 0.1 % sodium dodecyl sulfate, 0.1 % sodium deoxycholate)), Wash 2 buffer (20 mM HEPES (pH 7.4), 500 mM NaCl, 0.5 mM EDTA, 1.5 % Triton X-100, 0.1 % sodium deoxycholate, 10 % glycerol), and Wash 3 buffer (20 mM HEPES (pH 7.4), 300 mM NaCl, 1 mM MgCl_2_, 0.1 mM EDTA, 10 % glycerol, 1.5 % Triton X-100) for 10 min each. Bound proteins were eluted by incubation with 25 μl of Laemmli buffer (Bio-Rad) for 15 min at room temperature.

### Western blotting

Western blot analysis was performed in accordance with standard protocols [96]. For whole-cell protein extract preparation, 1.5 × 10^6^ cells were pelleted by centrifugation at 1250*g* and lysed in 100 μl of Laemmli sample buffer (Bio-Rad). Protein concentrations were determined using the Bio-Rad DC protein assay reagent in accordance with the manufacturer’s protocol. 15–20 μg of whole-cell protein extracts, 10 μl of nuclear extracts, or entire eluate from immunoprecipitated samples were separated on an 8 % SDS–polyacrylamide gel and transferred to a polyvinylidene difluoride (PVDF) membrane (Thermo Scientific) and subjected to western blot analysis. Membranes were probed with various rabbit primary antibodies followed by incubation with donkey anti-rabbit HRP-conjugated IgG (1:3000; GE Healthcare, NA9340) secondary antibody where applicable. The antibody signals were detected using the ECL^+^ or ECL prime western blot detection system (GE Healthcare). Primary antibodies used: HA-HRP (1:6000; Roche, 2013189), SIN3 (1:2000; [21]), RPD3 (1:3000; [21]), dKDM5/LID (1:5000; kindly provided by Dr. Julie Secombe [32]), beta-actin (1:1000; Cell Signaling, 4967).

### RNA interference

RNAi was performed based on modification of a published protocol [97]. In brief, 4 × 10^6^ cells were plated in 4 ml of Schneider’s *Drosophila* medium in a 60-mm diameter dish. After 3 h, FBS-containing medium was removed and replaced with 2 ml of serum-free medium. 50 μg of dsRNA was added per dish and mixed by swirling. After 30 min, 4 ml of Schneider’s *Drosophila* medium was added. Cells were assayed four days following the addition of dsRNA. RNAi was performed using dsRNA corresponding to *Sin3A* or *lid* mRNA. Construction of the *Sin3A* RNAi targeting sequence in pCRII-Topo vector and production of dsRNA is previously described [17]. The sequences in the pCRII-Topo vector for *lid* knockdown were generated using the following primer set 5′ to 3′ (forward primer) CGA CAT GGC CGA AAT GGT and (reverse primer) GAT ACC CAG TTG CTG TAT GAC. dsRNA against *GFP* was used as a control. PCR templates for targeting the *GFP* gene were generated from template DNA (kindly provided by Dr. Russell L. Finley, Jr.) using the following T7 promoter sequence containing primer set 5′ to 3′ (forward primer) GAA TTA ATA CGA CTC ACT ATA GGG AGA TGC CAT CTT CCT TGA AGT CA and (reverse primer) GAA TTA ATA CGA CTC ACT ATA GGG AGA TGA TGT TAA CGG CCA CAA GTT. Efficient knockdown of the target was routinely verified either at the protein level by western blotting or at the transcript level by real-time quantitative reverse transcription PCR (qRT-PCR).

### Cell proliferation assay

Mock (GFP dsRNA)-treated or RNAi-treated cells were stained with Trypan Blue and cells were counted 4 days after RNAi treatment. Cell density of each sample was calculated as per hemocytometer standards.

### Flow cytometry

10^6^ cells were pelleted and washed in 1 X PBS + 1 % BSA with centrifugation at 100*g* at room temperature for 5 min. Pelleted cells were resuspended in 100 μl of 1 X PBS + 4 % paraformaldehyde and incubated at room temperature for 15 min. Cells were then pelleted at 100*g*, the fixative discarded and resuspended in 1 ml 1 X PBS + 1 % BSA and stored at 4 °C. On the day of staining cells were pelleted by centrifugation at 100*g* at room temperature for 5 min, resuspended in 100 μl of Saponin permeabilization buffer and stained by the addition of 1 ml of dilute FxCycle Violet DNA stain (1:1000 in 1 X PBS) and incubated at room temperature for 30 min. Stained cells were analyzed in an Attune acoustic focusing cytometer (Applied Biosystems) using Attune software.

### Drosophila stocks

*Drosophila melanogaster* stocks were maintained, and crosses were performed, according to standard laboratory procedures. The following stocks were used: *UAS*-*SIN3*^*RNAi*-*I*^ [18], *SIN3 KD I* [28], and *SIN3 KD II* [52]; *Act*-*GAL4* (4414), *Ser*-*GAL4* (6791), *Bx*-*GAL4* (8696), *En*-*GAL4* (8828), *UAS*-*LID*^*RNAi*-*TRiP*^ (28,944), and *UAS*-*mCherry*^*RNAi*-*TRiP*^ (35,785), obtained from the Bloomington Stock Center; *UAS*-*LID*^*RNAi*-*KK*^ (103,830) obtained from Vienna *Drosophila* RNAi Center; *UAS*-*LID* and *UAS*-*LID*-JmjC* [32] kindly provided by Dr. Julie Secombe; *hsFLP;Act5C* > *CD2* > *GAL4,UAS*-*EGFP* kindly provided by Dr. Dirk Bohmann.

### Imaging flies

Whole flies were imaged at 30× magnification using an Olympus DP72 camera coupled to an Olympus SZX16 microscope. Wings were imaged at 80× magnification using a SPOT RT color camera coupled to a Leica MZ125 microscope.

### GFP clonal analysis

*hsFLP;Act5C* > *CD2* > *GAL4,UAS*-*EGFP* flies were crossed to *mCherry*^*RNAi*-*TRiP*^, *UAS*-*SIN3*^*RNAi*-*I*^, *UAS*-*LID*^*RNAi*-*TRiP*^, or *UAS*-*LID*^*RNAi*-*KK*^ to generate random GFP-positive clones. 0–4 h embryos were collected and heat shocked for 2 h at 37 °C, 48–52 h after egg laying. Wing discs from wandering third instar larvae were dissected and immunostained with antibody against GFP as described below.

### Immunostaining

Wing discs from wandering third instar larvae were dissected in 1 X PBS. Roughly 50 wing discs were fixed in 4 % formaldehyde in 1 X PBS and stained as previously described [18]. Antibody against GFP (1:1000; Abcam, ab1218) followed by sheep anti-mouse Alexa 488 (1:2000; Life Technologies, A11001) was used for staining. Visualization and imaging was done using a Zeiss Axioscope 2 fitted with an Axio-photo photography system.

### Gene expression analysis by RNAseq

RNA isolation to next-generation sequencing and initial quality control was performed at the Applied Genomics Technology Center, Wayne State University.

#### RNA isolation

S2 cells were subjected to RNAi alone or RNAi and 24 h treatment with 8.3 mM paraquat (1,1′-dimethyl-4,4′-bipyridinium dichloride (Sigma Aldrich)). Total RNA was isolated using the EZ1^®^ RNA Universal Tissue Kit (Qiagen). Cells were disrupted and homogenized in 750 µl QIAzol™ lysis reagent via bead-milling on the TissueLyser^®^ II (Qiagen). RNA was collected from the homogenate by chloroform extraction and purified on the EZ1^®^ Advanced (Qiagen) with additional DNase step to remove any residual DNA. Purified total RNA was quantified by UV spectrophotometry using the DropSense96^®^ Microplate Spectrophotometer (Trinean) and purity was assessed based on the A260/A280 and A260/A230 ratios. RNA quality was assessed by microfluidics using the RNA R6K assay for the Agilent 2200 TapeStation. The electrophoretogram was examined to determine overall quality of the RNA.

#### Next-generation sequencing

The TruSeq RNA Sample Preparation Kit (Illumina) was used to prepare adapter ligated PCR fragments for sequencing. In brief, mRNA was purified from total RNA and fragmented. The cleaved mRNA was primed with random hexamers and reverse transcribed into first strand cDNA. The RNA template was then removed and a replacement, complementary strand was generated. The ends of the double-stranded cDNA was repaired and adenylated. Then sequencing adapters were ligated to the prepared cDNA. PCR was used to selectively enrich the fragments containing the adapters. The PCR fragments were validated using Agilent 2200 TapeStation. Single indexed samples were multiplexed and sequenced on an Illumina HiSeq 2500 sequencing system in paired-end mode with a read length of 2 × 50 bp. Samples were demultiplexed using Illumina bcl2fastq converter (v1.8.3). Read quality was assessed with FastQC. The RNAseq experiments were conducted in triplicate. Depth of coverage of ~25–30 million reads was obtained.

#### Bioinformatic analysis

The Tuxedo pipeline was utilized for analysis [98]. The high-performance GRID computing system at Wayne State University was used for the Tuxedo pipeline analysis. In brief, reads obtained from RNA sequencing were aligned to the UCSC reference genome (dm3) using Bowtie/Tophat. Cufflinks was used to assemble the aligned reads into transcripts. The obtained reads were mapped to a total of 14,542 Refseq genes and FPKM (fragments per kilobase per million fragments mapped) values reflecting mRNA expression levels were generated through Cufflinks. Cuffdiff, an integrated package of Cufflinks, was used to identify statistically significant genes that were differentially expressed in treatment conditions compared to control. The default false discovery rate (FDR) of 5 % was used for the differential expression analysis. The R statistics environment was used to visualize the data. The correlation plots were generated using the Lattice package. The volcano plots were generated using the CummeRbund package. The heatmap was generated using gplots package (heatmap.2). Gene ontology analysis was performed using DAVID [54]. The functional annotation tool of DAVID was utilized where gene ontology term, GOTERM_BP_FAT was utilized to identify enriched biological processes and KEGG_PATHWAY was used to identify enriched pathways. The RNAseq data are available in the NCBI gene expression omnibus (GEO) database under accession number GSE68775 [99].

### Gene expression analysis by real-time quantitative RT-PCR

Total RNA was extracted from 1 × 10^7^ S2 cells subjected to RNA interference and paraquat treatment using the RNeasy mini kit (Qiagen). Extracted total RNA was used to generate cDNA with random hexamers using the ImProm-II Reverse Transcription System (Promega). Generated cDNA was used as the template in a real-time quantitative PCR assay carried out in a stratagene MX3005P real-time thermocycler. Analysis was performed using Absolute SYBR green ROX master mix (Fisher Scientific). Relative fold change in gene expression was determined by the comparative quantification (2^−ΔΔCT^) method of analysis [100]. *Taf1* was used to normalize cDNA amounts in the comparative analysis. The primer sets used in the PCR reactions are listed in Additional file 4: Table S1.

### Statistical analyses

All significance values were calculated by the two sample Student’s *t* test using GRAPHPAD. http://www.graphpad.com/quickcalcs/index.cfm.

### Chromatin immunoprecipitation and real-time quantitative PCR

4 × 10^7^ cells were crosslinked with 1 % formaldehyde for 10 min and quenched with 125 mM glycine. The cells were then pelleted and washed three times with 1 X PBS, with centrifugation each time at 1250*g* at 4 °C for 5 min. The obtained pellet was resuspended in 15 ml of resuspension buffer (10 mM Tris (pH 8), 10 mM KCl, 3 mM CaCl_2_, 0.34 M Sucrose, 1 mM DTT, 0.1 % Triton X-100, 0.2 mM EGTA, 1 Roche complete protease inhibitor tablet) and incubated on ice for 15 min. The resuspended cells were then homogenized by a dounce homogenizer using a loose pestle ten times and a tight pestle 15 times. The homogenized cells were pelleted at 170*g* at 4 °C for 10 min. The pellet was then resuspended in 200 μl of 10X MNase digest buffer (15 mM Tris (pH 8), 60 mM KCl, 15 mM NaCl, 1 mM CaCl_2_, 0.25 M sucrose, 1 mM DTT), and subjected to MNase digestion using 20 units of MNase for 10 min at room temperature. 10 mM EDTA was added to stop the reaction. Samples were diluted with NaCl buffer (140 mM NaCl, 10 mM Tris (pH 7.6), 2 mM EDTA) to a final volume of 1.2 ml and subjected to sonication for seven 30 s pulses with 1 min intervals at 20 % amplitude using an Ultrasonic dismembrator [Model 500 (Fisher Scientific)] sonicator. Sonicated samples were subjected to centrifugation at 15,000*g* for 15 min at 4 °C and the pellet was discarded. 75 μg of prepared chromatin was diluted to a final volume of 500 μl with NaCl buffer and used for immunoprecipitation. For immunoprecipitation of HA-tagged proteins 30 μl of anti-HA beads (monoclonal anti-HA agarose conjugate clone HA-7 (Sigma, A2095)) were added to 500 μl of prepared chromatin samples and placed on a nutator at 4 °C for 4 h. For immunoprecipitation of native protein or modified histones antibody specific to SIN3 [21] (5 μg), H3 C-terminus (Abcam, ab1791), H3K9Ac (Millipore, 07-352), H3K4Me3 (Active Motif, 39,159), or IgG (2.5 μg) as control was added to 500 μl of prepared chromatin samples and placed on a nutator at 4 °C overnight. 30 μl of anti-IgG beads [Protein A agarose (Pierce)] were then added to antibody treated chromatin samples and the tubes were placed on a nutator at 4 °C for 4 h. Anti-HA or anti-IgG beads were then washed with Wash 1 buffer (50 mM Tris (pH 7.6), 280 mM NaCl, 2 mM EDTA, 0.3 % sodium dodecyl sulfate), Wash 2 buffer (25 mM Tris (pH 7.6), 500 mM NaCl, 1 mM EDTA, 0.1 % sodium deoxycholate, 1 % Triton X-100), and Wash 3 buffer (10 mM Tris (pH 7.6), 250 mM LiCl, 1 mM EDTA, 0.5 % sodium deoxycholate, 0.5 % Triton X-100) for 10 min each at 4 °C. Finally beads were rinsed with Tris–EDTA (pH 8.0) and eluted with 500 μl of elution buffer (1 % sodium dodecyl sulfate, 0.1 M NaHCO_3_) at 65 °C for 1 h. Eluted samples were treated with 0.05 μg/μl RNase A at 37 °C for 15 min and DNA:protein crosslinks were reversed by overnight incubation at 65 °C after addition of 200 mM NaCl. Samples were Proteinase K treated [0.04 μM Proteinase K, 10 μM EDTA, 20 μM Tris (pH 7.5)] at 45 °C for 1.5 h and subjected to phenol chloroform extraction and ethanol precipitation. Precipitated DNA was resuspended in 50 μl of ddH_2_0. Input DNA was prepared from 75 μg of chromatin samples directly after RNase treatment and reversal of crosslinks as described above. Input DNA (diluted 1:100) and immunoprecipitated samples (diluted 1:4) were subjected to real-time quantitative PCR with Absolute SYBR Green ROX master mix (Fisher Scientific) using a Stratagene MX3005P real-time thermocycler. The primer sets used in the PCR reactions are listed in Additional file 4: Table S2.

## Availability of supporting data

The datasets supporting the results of this article are available in NCBI Gene Expression Omnibus (GEO) database under accession number GSE68775 at http://www.ncbi.nlm.nih.gov/geo/query/acc.cgi?acc=GSE68775.

## Additional files

10.1186/s13072-016-0053-9 RNAi in S2 cells leads to efficient knockdown of *Sin3A* and *lid*. **Figure S2.** Wing developmental defects observed upon reduction or overexpression of dKDM5/LID in male flies. **Figure S3.** Biological replicates of RNAseq data correlate significantly. **Figure S4.** Gene expression changes upon knockdown of *Sin3A*, *lid* or both in S2 cells as determined by RNAseq. **Figure S5.** QPCR validates RNAseq data. **Figure S6.** Flow cytometry analysis of S2 cells knocked down for *Sin3A*, *lid* or both under oxidative stress conditions. **Figure S7.** SIN3 directly binds the TSS of many gene targets.
10.1186/s13072-016-0053-9 Differential gene expression analysis. Output from Cuffdiff analysis.
10.1186/s13072-016-0053-9 Gene ontology analysis. Summarized output from GO analysis using DAVID.
10.1186/s13072-016-0053-9 Primers used for gene expression analysis. **Table S2.** Primers used for ChIP-qPCR analysis.

## Abbreviations

HDAC: histone deacetylase
KDM: lysine demethylase
KAT: lysine acetyltransferase
RNAi: RNA interference
MEF: mouse embryonic fibroblast
qRT-PCR: quantitative reverse transcription PCR
DAVID: database for annotation, visualization, and integrated discovery
GO: gene ontology
ROS: reactive oxygen species
ChIP-qPCR: chromatin immunoprecipitation-quantitative PCR

## References

[1] Felsenfeld G, Groudine M (2003). Controlling the double helix. Nature.

[2] Bannister AJ, Kouzarides T (2011). Regulation of chromatin by histone modifications. Cell Res.

[3] Hayakawa T, Nakayama J (2011). Physiological roles of class I HDAC complex and histone demethylase. J Biomed Biotechnol.

[4] Silverstein RA, Ekwall K (2005). Sin3: a flexible regulator of global gene expression and genome stability. Curr Genet.

[5] Hayakawa T, Ohtani Y, Hayakawa N, Shinmyozu K, Saito M, Ishikawa F, Nakayama J (2007). RBP2 is an MRG15 complex component and down-regulates intragenic histone H3 lysine 4 methylation. Genes Cells.

[6] Moshkin YM, Kan TW, Goodfellow H, Bezstarosti K, Maeda RK, Pilyugin M, Karch F, Bray SJ, Demmers JA, Verrijzer CP (2009). Histone chaperones ASF1 and NAP1 differentially modulate removal of active histone marks by LID-RPD3 complexes during NOTCH silencing. Mol Cell.

[7] Spain MM, Caruso JA, Swaminathan A, Pile LA (2010). Drosophila SIN3 isoforms interact with distinct proteins and have unique biological functions. J Biol Chem.

[8] van Oevelen C, Wang J, Asp P, Yan Q, Kaelin WG, Kluger Y, Dynlacht BD (2008). A role for mammalian Sin3 in permanent gene silencing. Mol Cell.

[9] Cowley SM, Iritani BM, Mendrysa SM, Xu T, Cheng PF, Yada J, Liggitt HD, Eisenman RN (2005). The mSin3A chromatin-modifying complex is essential for embryogenesis and T-cell development. Mol Cell Biol.

[10] Dannenberg JH, David G, Zhong S, van der Torre J, Wong WH, Depinho RA (2005). mSin3A corepressor regulates diverse transcriptional networks governing normal and neoplastic growth and survival. Genes Dev.

[11] David G, Grandinetti KB, Finnerty PM, Simpson N, Chu GC, Depinho RA (2008). Specific requirement of the chromatin modifier mSin3B in cell cycle exit and cellular differentiation. Proc Natl Acad Sci USA.

[12] Neufeld TP, Tang AH, Rubin GM (1998). A genetic screen to identify components of the sina signaling pathway in Drosophila eye development. Genetics.

[13] Pennetta G, Pauli D (1998). The Drosophila Sin3 gene encodes a widely distributed transcription factor essential for embryonic viability. Dev Genes Evol.

[14] Nasmyth K, Stillman D, Kipling D (1987). Both positive and negative regulators of HO transcription are required for mother-cell-specific mating-type switching in yeast. Cell.

[15] Sternberg PW, Stern MJ, Clark I, Herskowitz I (1987). Activation of the yeast HO gene by release from multiple negative controls. Cell.

[16] Pile LA, Spellman PT, Katzenberger RJ, Wassarman DA (2003). The SIN3 deacetylase complex represses genes encoding mitochondrial proteins: implications for the regulation of energy metabolism. J Biol Chem.

[17] Pile LA, Schlag EM, Wassarman DA (2002). The SIN3/RPD3 deacetylase complex is essential for G(2) phase cell cycle progression and regulation of SMRTER corepressor levels. Mol Cell Biol.

[18] Sharma V, Swaminathan A, Bao R, Pile LA (2008). Drosophila SIN3 is required at multiple stages of development. Dev Dyn.

[19] Barnes VL, Bhat A, Unnikrishnan A, Heydari AR, Arking R, Pile LA (2014). SIN3 is critical for stress resistance and modulates adult lifespan. Aging.

[20] Tsai CC, Kao HY, Yao TP, McKeown M, Evans RM (1999). SMRTER, a *Drosophila* nuclear receptor coregulator, reveals that EcR-mediated repression is critical for development. Mol Cell.

[21] Pile LA, Wassarman DA (2000). Chromosomal localization links the SIN3-RPD3 complex to the regulation of chromatin condensation, histone acetylation and gene expression. EMBO J.

[22] Mummery-Widmer JL, Yamazaki M, Stoeger T, Novatchkova M, Bhalerao S, Chen D, Dietzl G, Dickson BJ, Knoblich JA (2009). Genome-wide analysis of Notch signalling in Drosophila by transgenic RNAi. Nature.

[23] Friedman A, Perrimon N (2006). A functional RNAi screen for regulators of receptor tyrosine kinase and ERK signalling. Nature.

[24] Bond D, Foley E (2009). A quantitative RNAi screen for JNK modifiers identifies Pvr as a novel regulator of Drosophila immune signaling. PLoS Pathog.

[25] Kim YO, Park SJ, Balaban RS, Nirenberg M, Kim Y (2004). A functional genomic screen for cardiogenic genes using RNA interference in developing Drosophila embryos. Proc Natl Acad Sci USA.

[26] Parrish JZ, Kim MD, Jan LY, Jan YN (2006). Genome-wide analyses identify transcription factors required for proper morphogenesis of Drosophila sensory neuron dendrites. Genes Dev.

[27] Sepp KJ, Hong P, Lizarraga SB, Liu JS, Mejia LA, Walsh CA, Perrimon N (2008). Identification of neural outgrowth genes using genome-wide RNAi. PLoS Genet.

[28] Swaminathan A, Pile LA (2010). Regulation of cell proliferation and wing development by Drosophila SIN3 and String. Mech Dev.

[29] Gildea JJ, Lopez R, Shearn A (2000). A screen for new trithorax group genes identified little imaginal discs, the Drosophila melanogaster homologue of human retinoblastoma binding protein 2. Genetics.

[30] Eissenberg JC, Lee MG, Schneider J, Ilvarsonn A, Shiekhattar R, Shilatifard A (2007). The trithorax-group gene in Drosophila little imaginal discs encodes a trimethylated histone H3 Lys4 demethylase. Nat Struct Mol Biol.

[31] Lee N, Zhang J, Klose RJ, Erdjument-Bromage H, Tempst P, Jones RS, Zhang Y (2007). The trithorax-group protein Lid is a histone H3 trimethyl-Lys4 demethylase. Nat Struct Mol Biol.

[32] Secombe J, Li L, Carlos L, Eisenman RN (2007). The Trithorax group protein Lid is a trimethyl histone H3K4 demethylase required for dMyc-induced cell growth. Genes Dev.

[33] Lloret-Llinares M, Carre C, Vaquero A, de Olano N, Azorin F (2008). Characterization of Drosophila melanogaster JmjC + N histone demethylases. Nucleic Acids Res.

[34] Klose RJ, Yan Q, Tothova Z, Yamane K, Erdjument-Bromage H, Tempst P, Gilliland DG, Zhang Y, Kaelin WG (2007). The retinoblastoma binding protein RBP2 is an H3K4 demethylase. Cell.

[35] Barrett A, Santangelo S, Tan K, Catchpole S, Roberts K, Spencer-Dene B, Hall D, Scibetta A, Burchell J, Verdin E (2007). Breast cancer associated transcriptional repressor PLU-1/JARID1B interacts directly with histone deacetylases. Int J Cancer.

[36] Tahiliani M, Mei P, Fang R, Leonor T, Rutenberg M, Shimizu F, Li J, Rao A, Shi Y (2007). The histone H3K4 demethylase SMCX links REST target genes to X-linked mental retardation. Nature.

[37] Lee N, Erdjument-Bromage H, Tempst P, Jones RS, Zhang Y (2009). The H3K4 demethylase lid associates with and inhibits histone deacetylase Rpd3. Mol Cell Biol.

[38] Liu X, Greer C, Secombe J (2014). KDM5 Interacts with Foxo to Modulate Cellular Levels of Oxidative Stress. PLoS Genet.

[39] Lloret-Llinares M, Perez-Lluch S, Rossell D, Moran T, Ponsa-Cobas J, Auer H, Corominas M, Azorin F (2012). dKDM5/LID regulates H3K4me3 dynamics at the transcription-start site (TSS) of actively transcribed developmental genes. Nucleic Acids Res.

[40] Li L, Greer C, Eisenman RN, Secombe J (2010). Essential functions of the histone demethylase lid. PLoS Genet.

[41] Benevolenskaya EV, Murray HL, Branton P, Young RA, Kaelin WG (2005). Binding of pRB to the PHD protein RBP2 promotes cellular differentiation. Mol Cell.

[42] Lopez-Bigas N, Kisiel TA, Dewaal DC, Holmes KB, Volkert TL, Gupta S, Love J, Murray HL, Young RA, Benevolenskaya EV (2008). Genome-wide analysis of the H3K4 histone demethylase RBP2 reveals a transcriptional program controlling differentiation. Mol Cell.

[43] Beshiri ML, Holmes KB, Richter WF, Hess S, Islam AB, Yan Q, Plante L, Litovchick L, Gevry N, Lopez-Bigas N (2012). Coordinated repression of cell cycle genes by KDM5A and E2F4 during differentiation. Proc Natl Acad Sci USA.

[44] Scibetta AG, Santangelo S, Coleman J, Hall D, Chaplin T, Copier J, Catchpole S, Burchell J, Taylor-Papadimitriou J (2007). Functional analysis of the transcription repressor PLU-1/JARID1B. Mol Cell Biol.

[45] Yamane K, Tateishi K, Klose RJ, Fang J, Fabrizio LA, Erdjument-Bromage H, Taylor-Papadimitriou J, Tempst P, Zhang Y (2007). PLU-1 is an H3K4 demethylase involved in transcriptional repression and breast cancer cell proliferation. Mol Cell.

[46] Stephan O, Koch C (2009). Sin3 is involved in cell size control at Start in *Saccharomyces cerevisiae*. FEBS J.

[47] Duffy JB (2002). GAL4 system in *Drosophila*: a fly geneticist’s Swiss army knife. Genesis.

[48] Liefke R, Oswald F, Alvarado C, Ferres-Marco D, Mittler G, Rodriguez P, Dominguez M, Borggrefe T (2010). Histone demethylase KDM5A is an integral part of the core Notch-RBP-J repressor complex. Genes Dev.

[49] Zhang H, Li Y, Yang J, Tominaga K, Pereira-Smith OM, Tower J (2010). Conditional inactivation of MRG15 gene function limits survival during larval and adult stages of Drosophila melanogaster. Exp Gerontol.

[50] Curtis BJ, Zraly CB, Marenda DR, Dingwall AK (2011). Histone lysine demethylases function as co-repressors of SWI/SNF remodeling activities during Drosophila wing development. Dev Biol.

[51] Di Stefano L, Walker JA, Burgio G, Corona DF, Mulligan P, Naar AM, Dyson NJ (2011). Functional antagonism between histone H3K4 demethylases in vivo. Genes Dev.

[52] Swaminathan A, Barnes VL, Fox S, Gammouh S, Pile LA (2012). Identification of genetic suppressors of the Sin3A knockdown wing phenotype. PLoS One.

[53] Brown EJ, Bachtrog D (2014). The chromatin landscape of *Drosophila*: comparisons between species, sexes, and chromosomes. Genome Res.

[54] da Huang W, Sherman BT, Lempicki RA (2009). Systematic and integrative analysis of large gene lists using DAVID bioinformatics resources. Nat Protoc.

[55] Benevolenskaya EV (2007). Histone H3K4 demethylases are essential in development and differentiation. Biochem Cell Biol.

[56] Herranz H, Milan M (2008). Signalling molecules, growth regulators and cell cycle control in *Drosophila*. Cell Cycle.

[57] Neufeld TP, de la Cruz AF, Johnston LA, Edgar BA (1998). Coordination of growth and cell division in the *Drosophila* wing. Cell.

[58] Stork T, Engelen D, Krudewig A, Silies M, Bainton RJ, Klambt C (2008). Organization and function of the blood-brain barrier in *Drosophila*. J Neurosci.

[59] Auld VJ, Fetter RD, Broadie K, Goodman CS (1995). Gliotactin, a novel transmembrane protein on peripheral glia, is required to form the blood-nerve barrier in *Drosophila*. Cell.

[60] Yi P, Johnson AN, Han Z, Wu J, Olson EN (2008). Heterotrimeric G proteins regulate a noncanonical function of septate junction proteins to maintain cardiac integrity in *Drosophila*. Dev Cell.

[61] Barnes VL, Strunk BS, Lee I, Huttemann M, Pile LA (2010). Loss of the SIN3 transcriptional corepressor results in aberrant mitochondrial function. BMC Biochem.

[62] Akerfelt M, Morimoto RI, Sistonen L (2010). Heat shock factors: integrators of cell stress, development and lifespan. Nat Rev Mol Cell Biol.

[63] Tower J (2011). Heat shock proteins and *Drosophila* aging. Exp Gerontol.

[64] Wang MC, Bohmann D, Jasper H (2005). JNK extends life span and limits growth by antagonizing cellular and organism-wide responses to insulin signaling. Cell.

[65] Paik D, Jang YG, Lee YE, Lee YN, Yamamoto R, Gee HY, Yoo S, Bae E, Min KJ, Tatar M (2012). Misexpression screen delineates novel genes controlling *Drosophila* lifespan. Mech Ageing Dev.

[66] Pallardo FV, Markovic J, Garcia JL, Vina J (2009). Role of nuclear glutathione as a key regulator of cell proliferation. Mol Aspects Med.

[67] Bonilla E, Medina-Leendertz S, Villalobos V, Molero L, Bohorquez A (2006). Paraquat-induced oxidative stress in drosophila melanogaster: effects of melatonin, glutathione, serotonin, minocycline, lipoic acid and ascorbic acid. Neurochem Res.

[68] Bus JS, Gibson JE (1984). Paraquat: model for oxidant-initiated toxicity. Environ Health Perspect.

[69] St Pierre SE, Ponting L, Stefancsik R, McQuilton P, FlyBase C (2014). FlyBase 102–advanced approaches to interrogating FlyBase. Nucleic Acids Res..

[70] Bachmann A, Draga M, Grawe F, Knust E (2008). On the role of the MAGUK proteins encoded by Drosophila varicose during embryonic and postembryonic development. BMC Dev Biol.

[71] FlyBase-Curators, Members S-PP, Members IP. Gene Ontology annotation in FlyBase through association of InterPro records with GO terms. 2004.

[72] Larsson J, Zhang J, Rasmuson-Lestander A (1996). Mutations in the Drosophila melanogaster gene encoding S-adenosylmethionine synthetase [corrected] suppress position-effect variegation. Genetics.

[73] Limbach KJ, Wu R (1985). Characterization of two Drosophila melanogaster cytochrome c genes and their transcripts. Nucleic Acids Res.

[74] Saisawang C, Wongsantichon J, Ketterman AJ (2012). A preliminary characterization of the cytosolic glutathione transferase proteome from Drosophila melanogaster. Biochem J.

[75] Suzuki T, Terasaki M, Takemoto-Hori C, Hanada T, Ueda T, Wada A, Watanabe K (2001). Structural compensation for the deficit of rRNA with proteins in the mammalian mitochondrial ribosome. Systematic analysis of protein components of the large ribosomal subunit from mammalian mitochondria. J Biol Chem.

[76] Jacinto E, Hall MN (2003). Tor signalling in bugs, brain and brawn. Nat Rev Mol Cell Biol.

[77] Hernandez G, Altmann M, Sierra JM, Urlaub H, Diez del Corral R, Schwartz P, Rivera-Pomar R (2005). Functional analysis of seven genes encoding eight translation initiation factor 4E (eIF4E) isoforms in *Drosophila*. Mech Dev.

[78] Lee JH, Budanov AV, Park EJ, Birse R, Kim TE, Perkins GA, Ocorr K, Ellisman MH, Bodmer R, Bier E (2010). Sestrin as a feedback inhibitor of TOR that prevents age-related pathologies. Science.

[79] Miron M, Verdu J, Lachance PE, Birnbaum MJ, Lasko PF, Sonenberg N (2001). The translational inhibitor 4E-BP is an effector of PI(3)K/Akt signalling and cell growth in *Drosophila*. Nat Cell Biol.

[80] Cully M, Genevet A, Warne P, Treins C, Liu T, Bastien J, Baum B, Tapon N, Leevers SJ, Downward J (2010). A role for p38 stress-activated protein kinase in regulation of cell growth via TORC1. Mol Cell Biol.

[81] Kondo S, Perrimon N (2011). A genome-wide RNAi screen identifies core components of the G(2)-M DNA damage checkpoint. Sci Signal.

[82] Bronstein R, Levkovitz L, Yosef N, Yanku M, Ruppin E, Sharan R, Westphal H, Oliver B, Segal D (2010). Transcriptional regulation by CHIP/LDB complexes. PLoS Genet.

[83] Camp D, Currie K, Labbe A, van Meyel DJ, Charron F (2010). Ihog and Boi are essential for Hedgehog signaling in *Drosophila*. Neural Dev.

[84] Carreira VP, Soto IM, Mensch J, Fanara JJ (2011). Genetic basis of wing morphogenesis in Drosophila: sexual dimorphism and non-allometric effects of shape variation. BMC Dev Biol.

[85] Yao S, Lum L, Beachy P (2006). The ihog cell-surface proteins bind Hedgehog and mediate pathway activation. Cell.

[86] Zid BM, Rogers AN, Katewa SD, Vargas MA, Kolipinski MC, Lu TA, Benzer S, Kapahi P (2009). 4E-BP extends lifespan upon dietary restriction by enhancing mitochondrial activity in *Drosophila*. Cell.

[87] Wang HD, Kazemi-Esfarjani P, Benzer S (2004). Multiple-stress analysis for isolation of *Drosophila* longevity genes. Proc Natl Acad Sci USA.

[88] Hao X, Zhang S, Timakov B, Zhang P (2007). The Hsp27 gene is not required for *Drosophila* development but its activity is associated with starvation resistance. Cell Stress Chaperones.

[89] Celniker SE, Dillon LA, Gerstein MB, Gunsalus KC, Henikoff S, Karpen GH, Kellis M, Lai EC, Lieb JD, MacAlpine DM (2009). Unlocking the secrets of the genome. Nature.

[90] Filion GJ, van Bemmel JG, Braunschweig U, Talhout W, Kind J, Ward LD, Brugman W, de Castro IJ, Kerkhoven RM, Bussemaker HJ (2010). Systematic protein location mapping reveals five principal chromatin types in *Drosophila* cells. Cell.

[91] Gan Q, Schones DE, Ho Eun S, Wei G, Cui K, Zhao K, Chen X (2010). Monovalent and unpoised status of most genes in undifferentiated cell-enriched Drosophila testis. Genome Biol..

[92] Kharchenko PV, Alekseyenko AA, Schwartz YB, Minoda A, Riddle NC, Ernst J, Sabo PJ, Larschan E, Gorchakov AA, Gu T (2011). Comprehensive analysis of the chromatin landscape in *Drosophila* melanogaster. Nature.

[93] Blair LP, Cao J, Zou MR, Sayegh J, Yan Q (2011). Epigenetic regulation by lysine demethylase 5 (KDM5) enzymes in cancer. Cancers (Basel).

[94] Kadamb R, Mittal S, Bansal N, Batra H, Saluja D (2013). Sin3: insight into its transcription regulatory functions. Eur J Cell Biol.

[95] Yu C, Wan KH, Hammonds AS, Stapleton M, Carlson JW, Celniker SE (2011). Development of expression-ready constructs for generation of proteomic libraries. Methods Mol Biol.

[96] Sambrook J, Russell DW (2001). Molecular Cloning: A Laboratory Manual, Third.

[97] Clemens JC, Worby CA, Simonson-Leff N, Muda M, Maehama T, Hemmings BA, Dixon JE (2000). Use of double-stranded RNA interference in *Drosophila* cell lines to dissect signal transduction pathways. Proc Natl Acad Sci USA.

[98] Trapnell C, Roberts A, Goff L, Pertea G, Kim D, Kelley DR, Pimentel H, Salzberg SL, Rinn JL, Pachter L (2012). Differential gene and transcript expression analysis of RNA-seq experiments with TopHat and Cufflinks. Nat Protoc.

[99] Gajan A, Liu M, Saha N, Pile LA. Transcriptome analysis upon SIN3 or dKDM5/LID knockdown or both under normal and paraquat induced stress conditions in *Drosophila* S2 cultured cells. NCBI Gene Expression Omnibus. 2015. http://www.ncbi.nlm.nih.gov/geo/query/acc.cgi?acc=GSE68775.

[100] Livak KJ, Schmittgen TD (2001). Analysis of relative gene expression data using real-time quantitative PCR and the 2(-Delta Delta C(T)) Method. Methods.

