# Supplementary material for: The histone demethylase dKDM5/LID interacts with the SIN3 histone deacetylase complex and shares functional similarities with SIN3
Source: Epigenetics Chromatin. 2016 Feb 3;9:4. doi: 10.1186/s13072-016-0053-9 (PMC4740996; doi:10.1186/s13072-016-0053-9)
Supplement: Supplementary file 1 — 10.1186/s13072-016-0053-9 RNAi in S2 cells leads to efficient knockdown of Sin3A and lid. Figure S2. Wing developmental defects observed upon reduction or overexpression of dKDM5/LID in male flies. Figure S3. Biological replicates of RNAseq data correlate significantly. Figure S4. Gene expression changes upon knockdown of Sin3A, lid or both in S2 cells as determined by RNAseq. Figure S5. QPCR validates RNAseq data. Figure S6. Flow cytometry analysis of S2 cells knocked down for Sin3A, lid or both under oxidative stress conditions. Figure S7. SIN3 directly binds the TSS of many gene targets. [file 13072_2016_53_MOESM1_ESM.pdf]

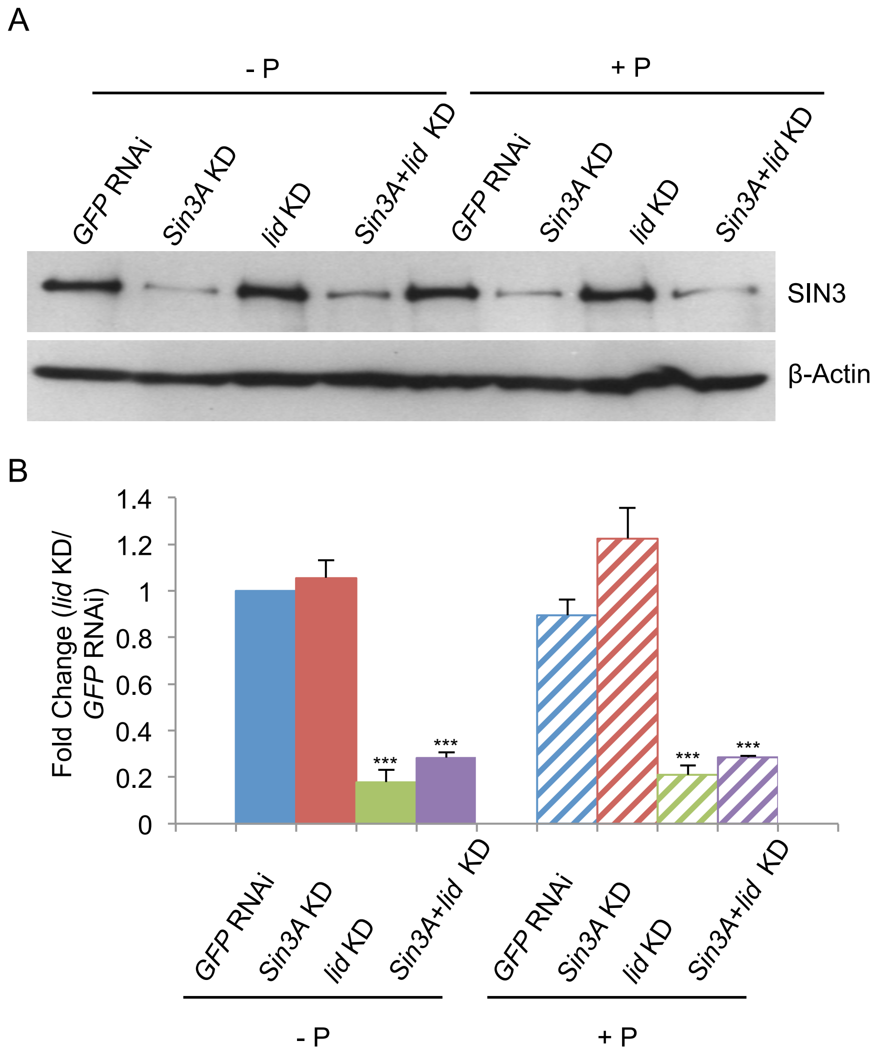

Figure S1. RNAi in S2 cells leads to efficient knockdown of *Sin3A* and *lid*. (A) Western blot analysis of whole cell protein extracts from cells treated with dsRNA targeting *Sin3A*, *lid* or both and *GFP* as a control. Blots were probed with antibody to SIN3 and β-actin as a loading control. (B) Real-time qRT-PCR analysis of *lid* transcript levels. *Taf1* was used to normalize expression levels. KD – knockdown, P – paraquat, ***, $P < 0.001$.

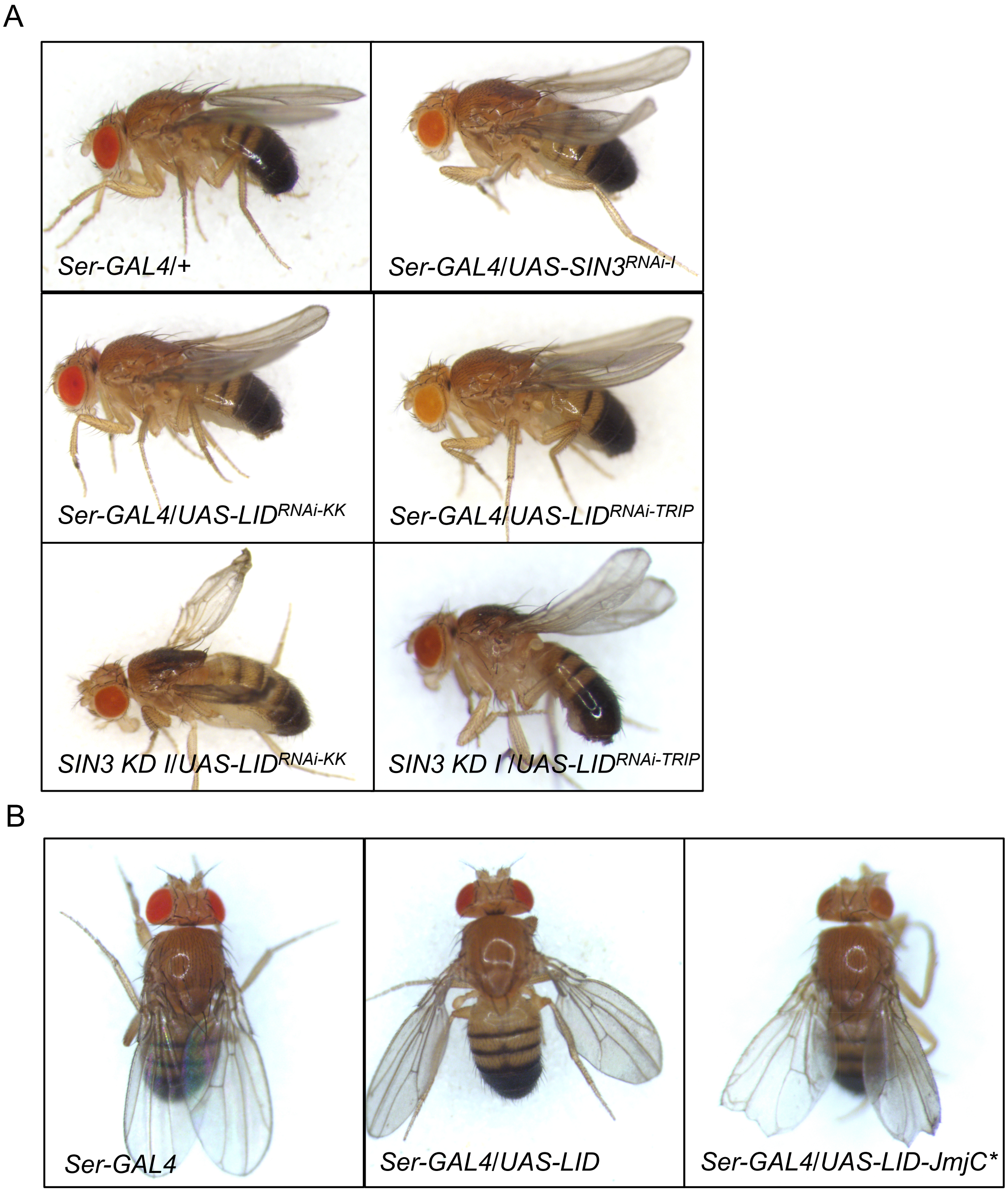

Figure S2*. Wing developmental defects observed upon reduction or overexpression of dKDM5/LID in male flies.* (A) Micrographs of male flies carrying the *Ser-GAL4* driver and/or the indicated UAS-RNAi constructs. *SIN3 KD I* flies carry both the *Ser-GAL4* driver and *UAS-SIN3^{RNAi}* construct. (B) Micrographs of male flies carrying the *Ser-GAL4* driver and the indicated UAS-overexpression constructs.

A

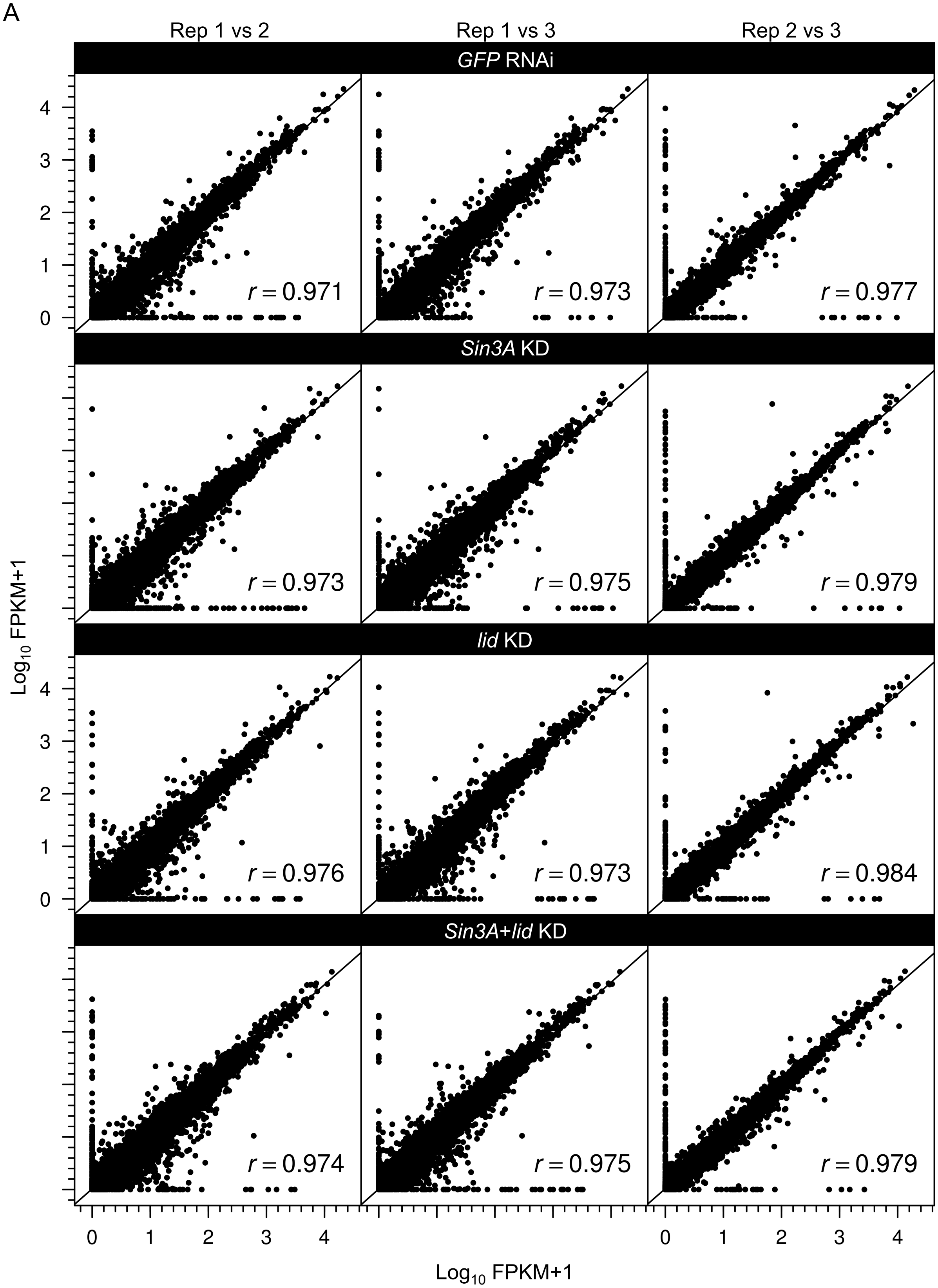

Rep 1 vs 2
Rep 1 vs 3
Rep 2 vs 3
GFP RNAi
Sin3A KD
lid KD
Sin3A+lid KD
r = 0.971
r = 0.973
r = 0.977
r = 0.973
r = 0.975
r = 0.979
r = 0.976
r = 0.973
r = 0.984
r = 0.974
r = 0.975
r = 0.979
0
1
2
3
4
Log10 FPKM+1
Log10 FPKM+1

B

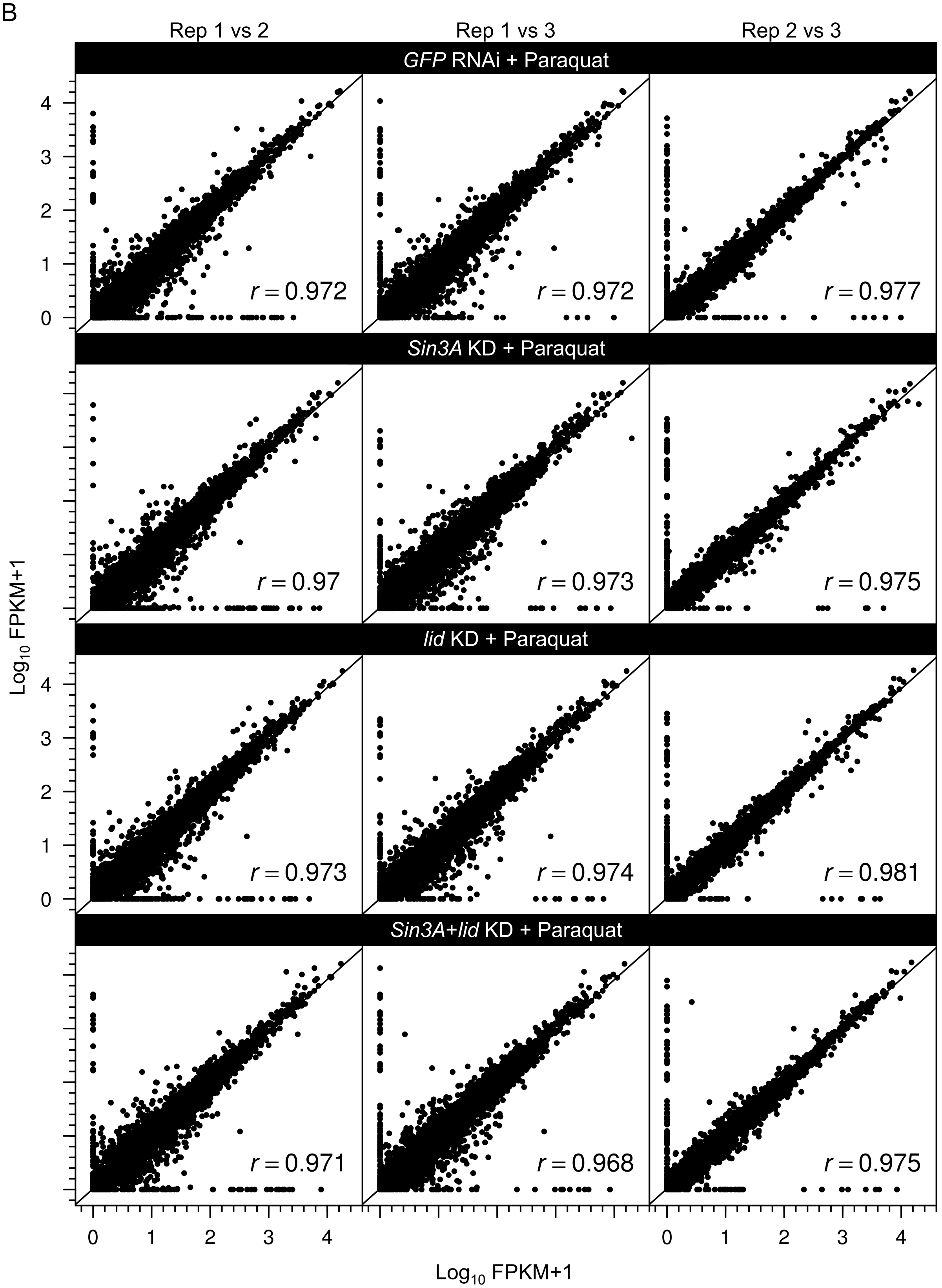

Figure S3. Biological replicates of RNAseq data correlate significantly. Correlation plots based on the FPKM values for the three replicates of RNAseq data from indicated samples under normal (A) and paraquat induced oxidative stress (B) conditions. The calculated Pearson correlation coefficients (*r*) for all datasets are significant, demonstrating reproducibility of the data. Rep – Replicate, KD - knockdown.

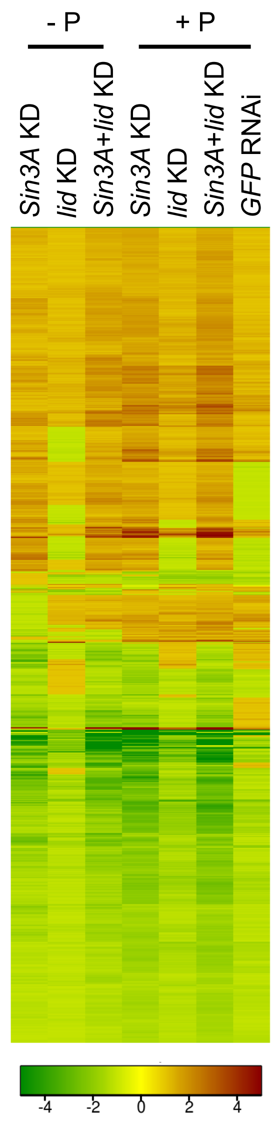

Figure S4. Gene expression changes upon knockdown of *Sin3A*, *lid* or both in S2 cells as determined by RNAseq. Heatmap plotting fold change in expression of genes from indicated samples compared to *GFP* RNAi treated controls. All genes significantly regulated upon at least one of the indicated conditions were hierarchically clustered, where genes showing similar trends across samples are clustered together, to generate the heatmap. The scale bar depicts fold change in expression. KD – knockdown, -P – untreated, +P – paraquat treated.

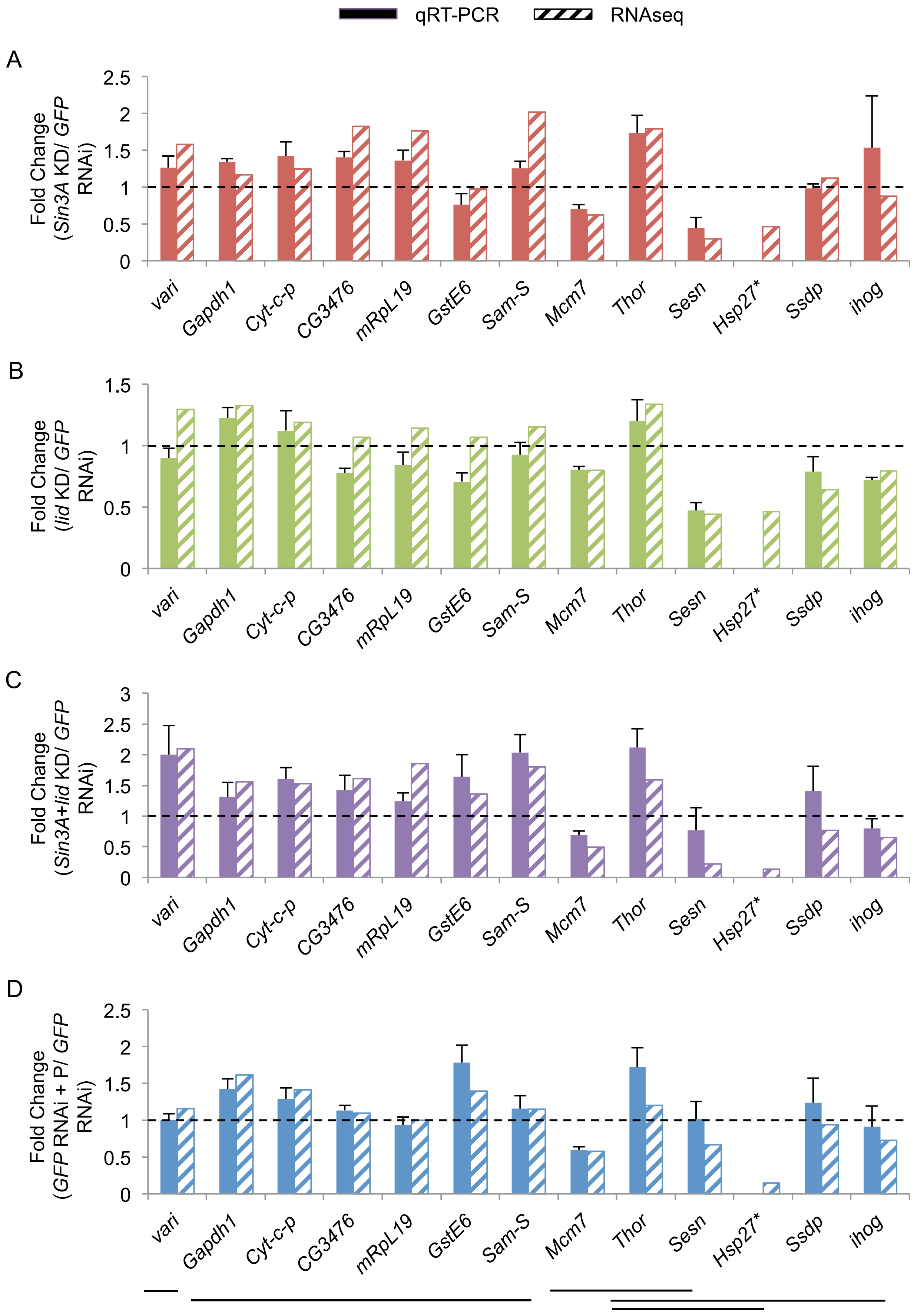

qRT-PCR
RNAseq
A
B
C
D
Fold Change (Sin3A KD/ GFP RNAi)
Fold Change (lid KD/ GFP RNAi)
Fold Change (Sin3A+lid KD/ GFP RNAi)
Fold Change (GFP RNAi + P/ GFP RNAi)
vari
Gapdh1
Cyt-c-p
CG3476
mRpL19
GstE6
Sam-S
Mcm7
Thor
Sesn
Hsp27*
Ssdp
ihog

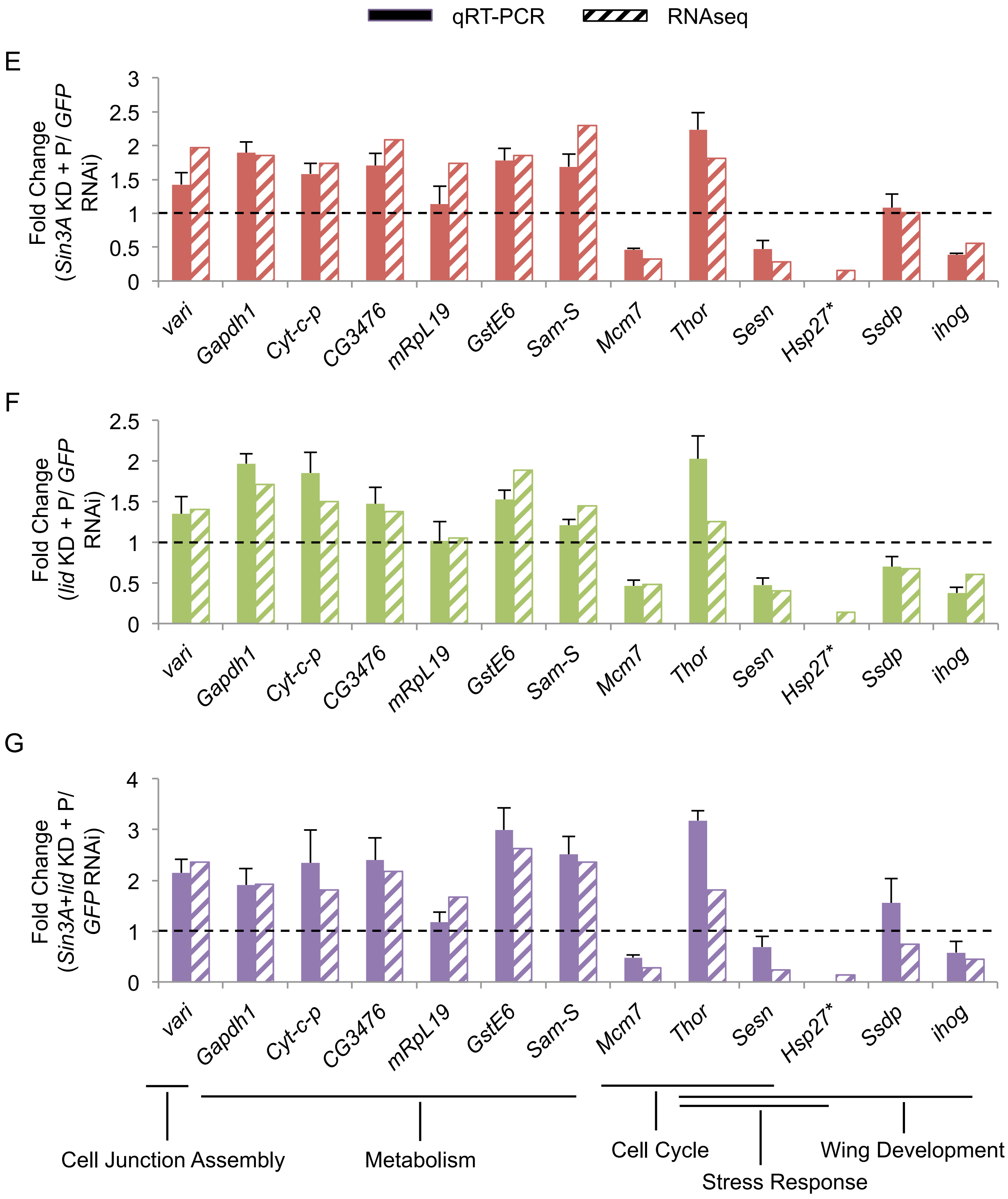

Figure S5. qPCR validates RNAseq data. Real time qRT-PCR analysis of total RNA extracts from S2 cells treated with dsRNA targeting indicated genes to induce knockdown (KD) under non-stressed conditions (A-C) or paraquat induced oxidative stress conditions (D-G). All experimental samples were compared to non-stressed *GFP* dsRNA treated controls. Primers targeting indicated genes were used for PCR amplification. *Taf1* was used to normalize expression levels. The results are the average of three - five biological replicates for qPCR and three biological replicates for RNAseq. Error bars represent standard error of the mean. KD – knockdown, P – paraquat, * - No qPCR data depicted for *Hsp27*.

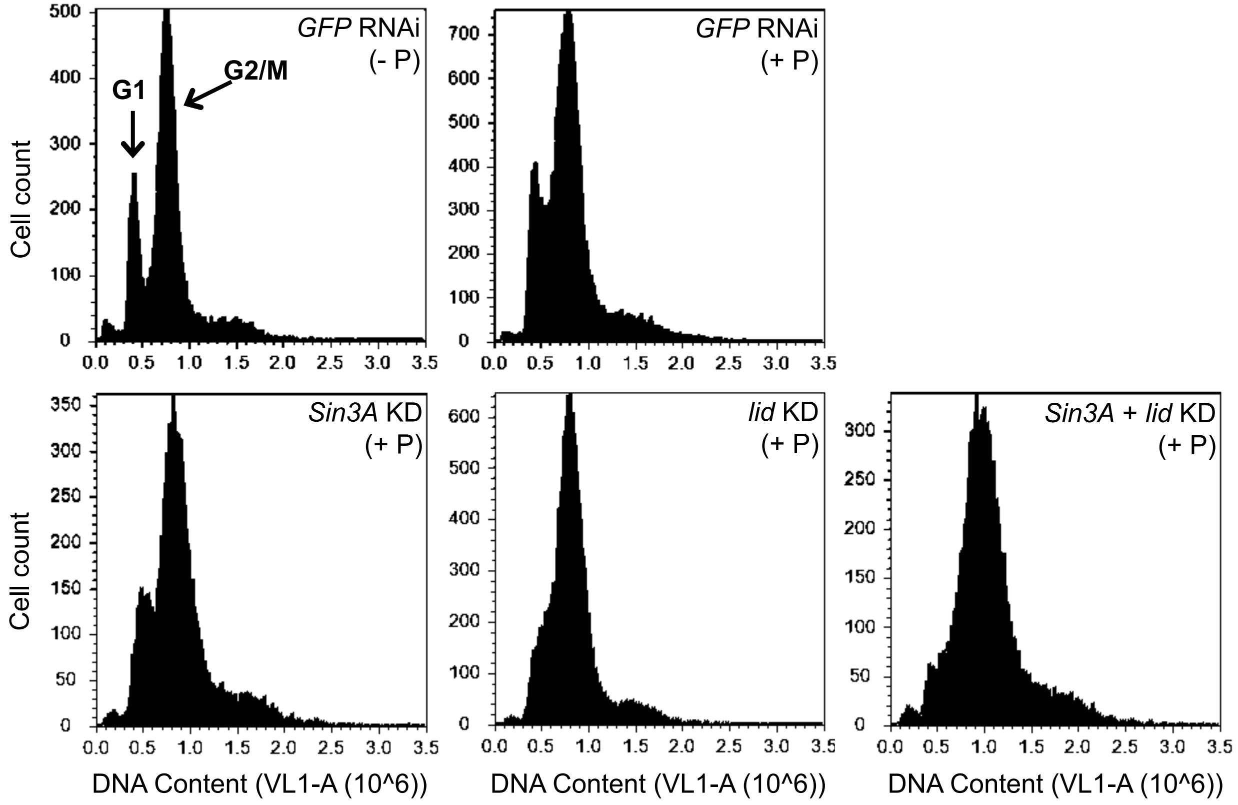

Figure S6. Flow cytometry analysis of S2 cells knocked down for *Sin3A*, *lid* or both under oxidative stress conditions. Histogram of DNA content vs. cell counts for indicated cell lines. Peaks for G1 and G2/M cell cycle phases are indicated in the top left panel. KD – knockdown, P – paraquat.

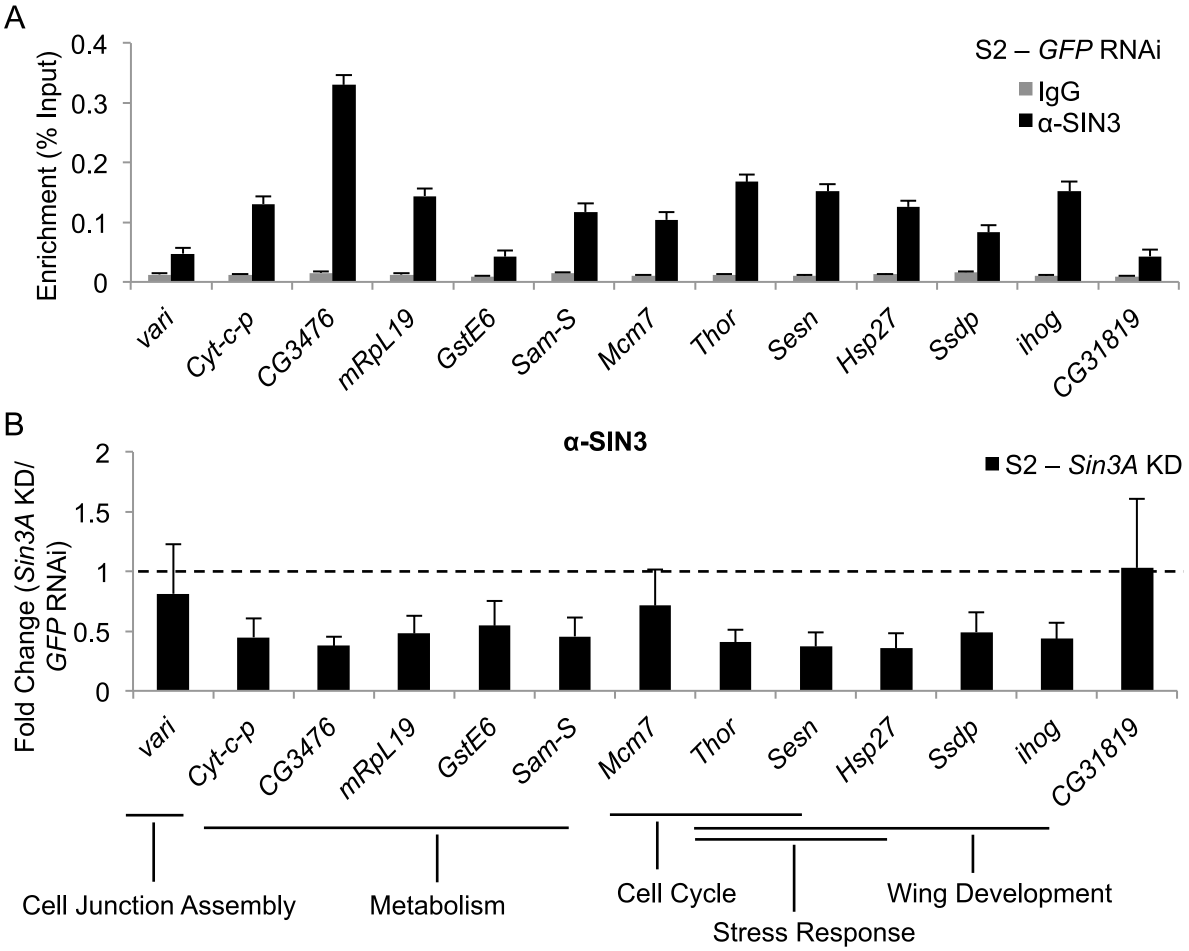

Figure S7. SIN3 directly binds the TSS of many gene targets. Real-time qPCR analysis of chromatin prepared from S2 cells immunoprecipitated with antibody to SIN3 or IgG as a control. (A) Enrichment of SIN3 at predicted target genes. (B) Fold change in enrichment of SIN3 upon knockdown of *Sin3A* at target genes. Primers used in the PCR amplification target regions spanning the TSS of indicated genes. *CG31819* acts as a negative control. The results are the average of three biological replicates. KD – knockdown.
