# Supplementary material for: The histone demethylase dKDM5/LID interacts with the SIN3 histone deacetylase complex and shares functional similarities with SIN3
Source: Epigenetics Chromatin. 2016 Feb 3;9:4. doi: 10.1186/s13072-016-0053-9 (PMC4740996; doi:10.1186/s13072-016-0053-9)
Supplement: Supplementary file 4 — 10.1186/s13072-016-0053-9 Primers used for gene expression analysis. Table S2. Primers used for ChIP-qPCR analysis. [file 13072_2016_53_MOESM4_ESM.pdf]

Table S1. Primers used for gene expression analysis. Listed 5' – 3'.

Gene	Forward Primer	Reverse Primer
lid	TCG TGC GAA AAG ACA CAG AA	GCC CGA TCT GCT TCA CCA GC
Taf1	CTG GTC CTG GTG AGG TGA	CCG GAT TCT GGG ATT TGA
vari	ACG CTC AAG AAT CGG CTG	ACG GCC TCT TCC ATT TCC
Gapdh1	GCC CTG AAC GGC AAG CT	GTA AGA TCC ACA ACG GAG ACA TTG
Cyt-c-p	GCT CGA CGT TTG TGT TCA AT	TTC CCT TCT CAA CAT CAC CA
CG3476	GCC ATC CAT TGG ACA CAA TA	GGC GGC ACA ATC TAT GAC T
mRpL19	CGA TCC CGA AAA CTA TTC AA	TTA CAT GTT CCG CAG TTT TG
GstE6	GAT CAG CGG CTG CAC TTT	TGG CAT CGT ATC GCT CCT
Sam-S	AAA CTT TGA CCT CAG GCC C	CGC TGG TAT ATC GGC TGG
Mcm7	ACC AAA TCC ACG AGC ACC	GGT CGG GCT TGA ATC CTT
Thor	GCT AAG ATG TCC GCT TCA CC	CCC GCT CGT AGA TAA GTT TGG
Sesn	GAG GAG CTC CAC CGG ACT	ATG CGC TCC ATT AGC GTC
Ssdp	GGA TTC CTG CAC ACC TGG	CCG TAG CCG GAG CTA ACA
ihog	GGA GGG GGC ACT GAA AAT	TAA CTG CGC AAA CGC AAA

Table S2. Primers used for ChIP-qPCR analysis. Listed 5' – 3'.

Gene	Forward Primer	Reverse Primer
**Transcription Factor Binding**		
vari	CCC AAC AAA GAA GTG GCG	TCC AAC AGC GCA AAA ACA
Cyt-c-p	GCA AAT TTT CCA GAG GCT TTC	GCC GAT TTT TCA CGA ATG AC
CG3476	CTG CAA TCG ATA GCT GAA TGT	GCG CGG TAT TAT AAT TTC CAT
mRpL19	TGG CAG TAC CCT TCC AAT TAT	ACA CAC TGC TGT GTC AAC CTA TT
GstE6	TTT TCT CTT TCA TTG ATC CCA A	GAC TGG GGT CCA AAC CGT
Sam-S	TTG AAC GCA GGT TGA GCA	CGC TCC GGA GTG AAC TGT
Mcm7	ACA CCT TTG GCA AGC AGC	ATT CCG CCA GAT CAT CCA
Thor	CGA GAG AGC AGG CGA AAG	TGT GTT CAC CGT TGG CTG
Sesn	TCG TTG CGA TTC GTT TCA	CGC TTT TCT AGC CGG ACA
Hsp27	CCT GGT TGC CAT GCA CTA	TGC TTC AAC GTT TGC CTT C
Ssdp	CAC TGA AAA TGG CGT GCT T	AAG TTG CGT CGT CGT CGT
ihog	TCC ACT GTA CCG CGA TGA	GGG ATG CTG GAA CTG GAA
CG31819	AGC GCT GCC AGA AGA AGA	GGT CAA AGT CTC CCA ATT TTC A
**Histone Modifications**		
CG3476	GCG CTA TTC GGA ATC ACA	ATC GCT GGC TTC CTG ATG
mRpL19	TTT GTT TCA GCC GAT CCC	CGT TTC CTG AAT GTG TGG C
Sesn	GAA AAC GGA CGA AAA TCG AG	CAC GAA AAC TGT GGA TAA AAT G
Ssdp	CAG CCG AAC AGG CAG TAA A	CGC TGT GTT GTT GCT GCT
wb	GCG ATC CAC AAG GTC CAC	TAC GCA AAC GAC CGT CAA
